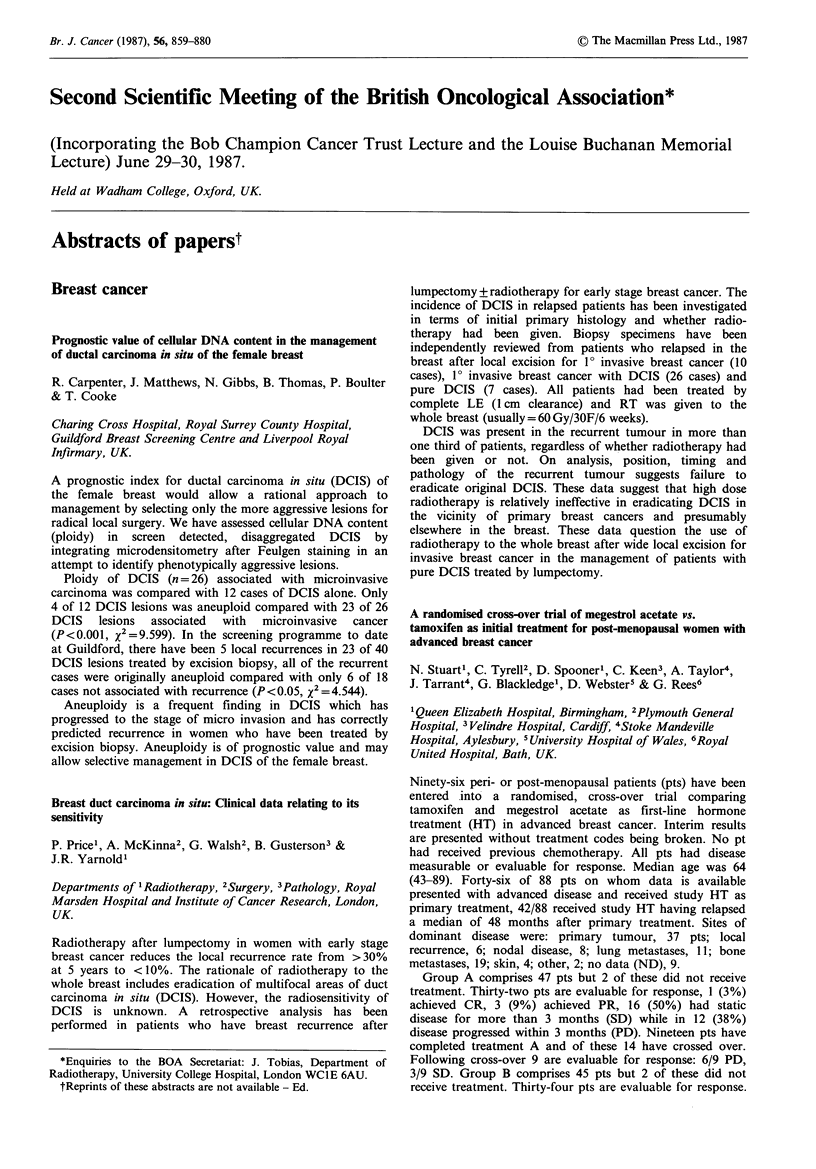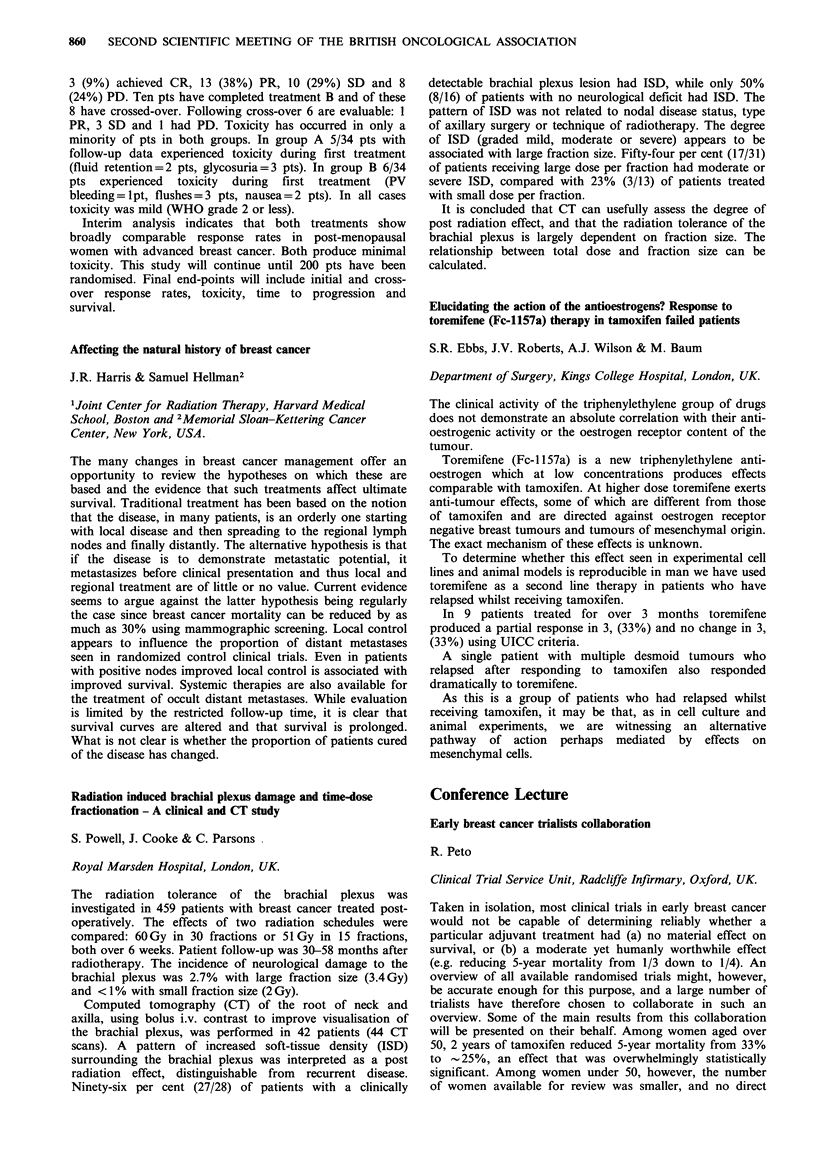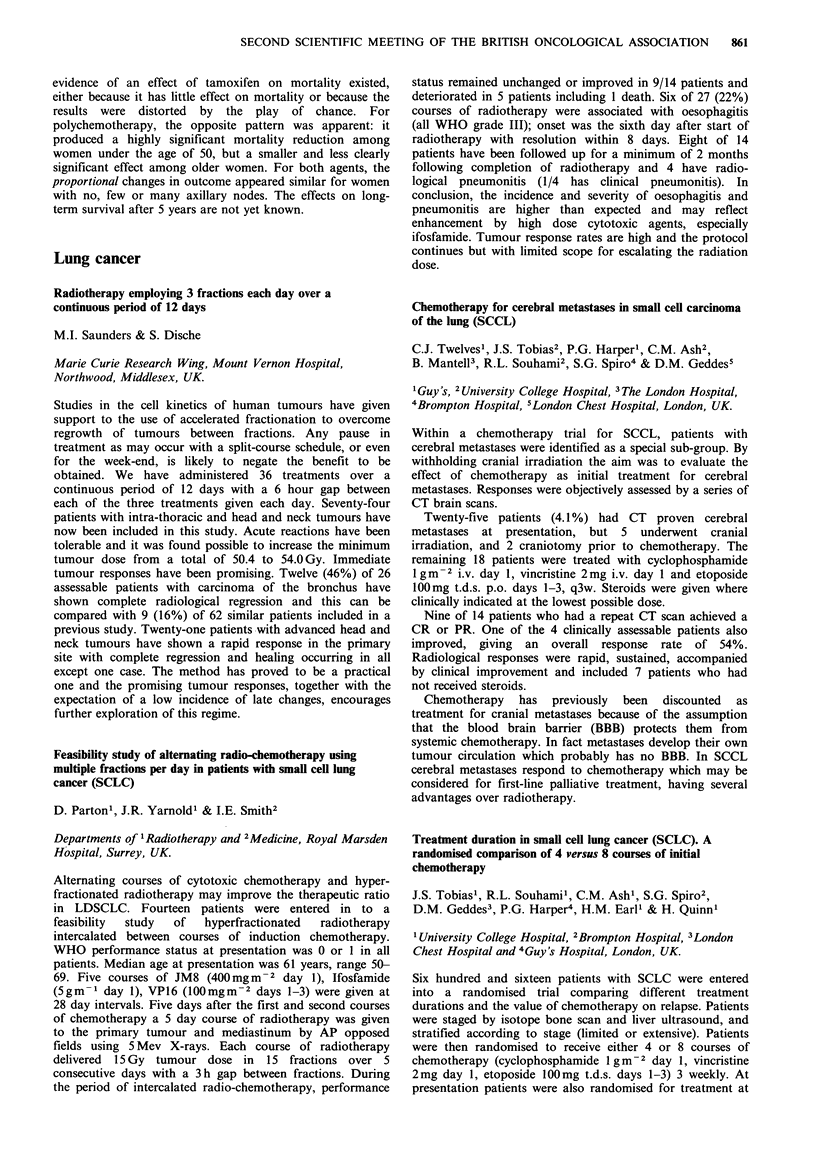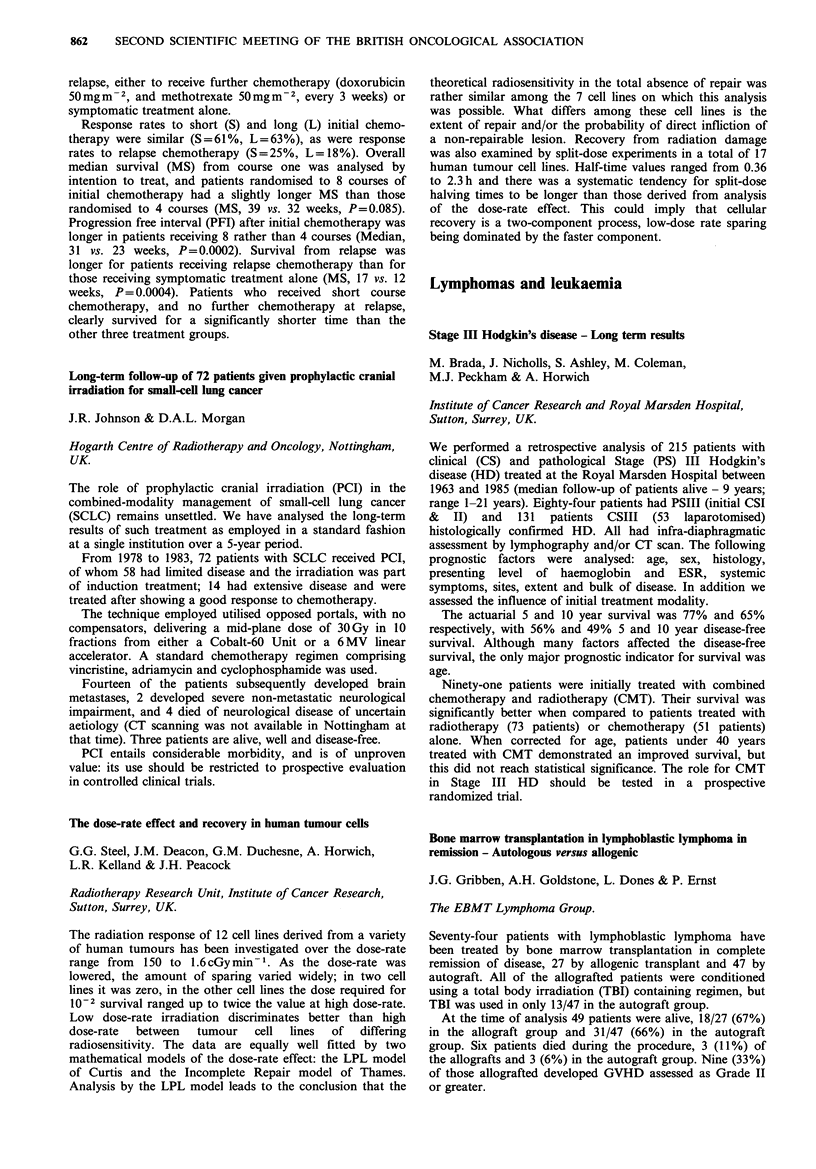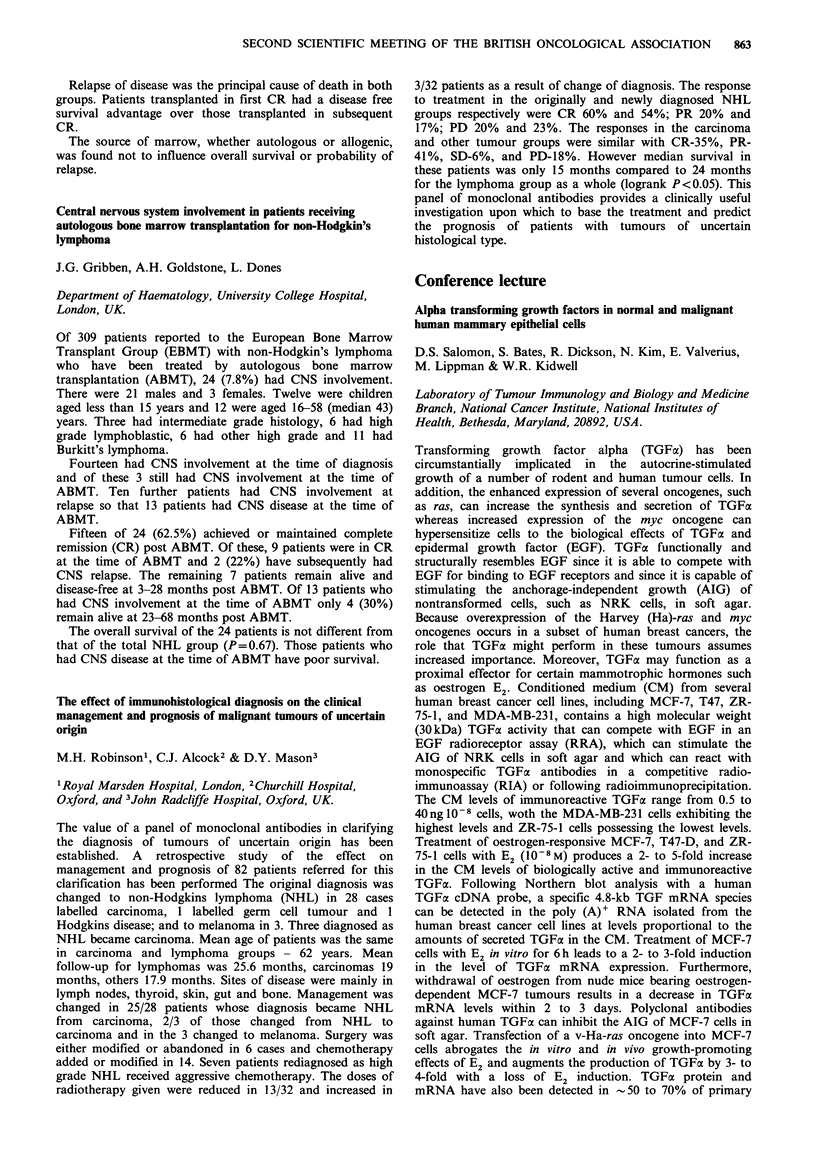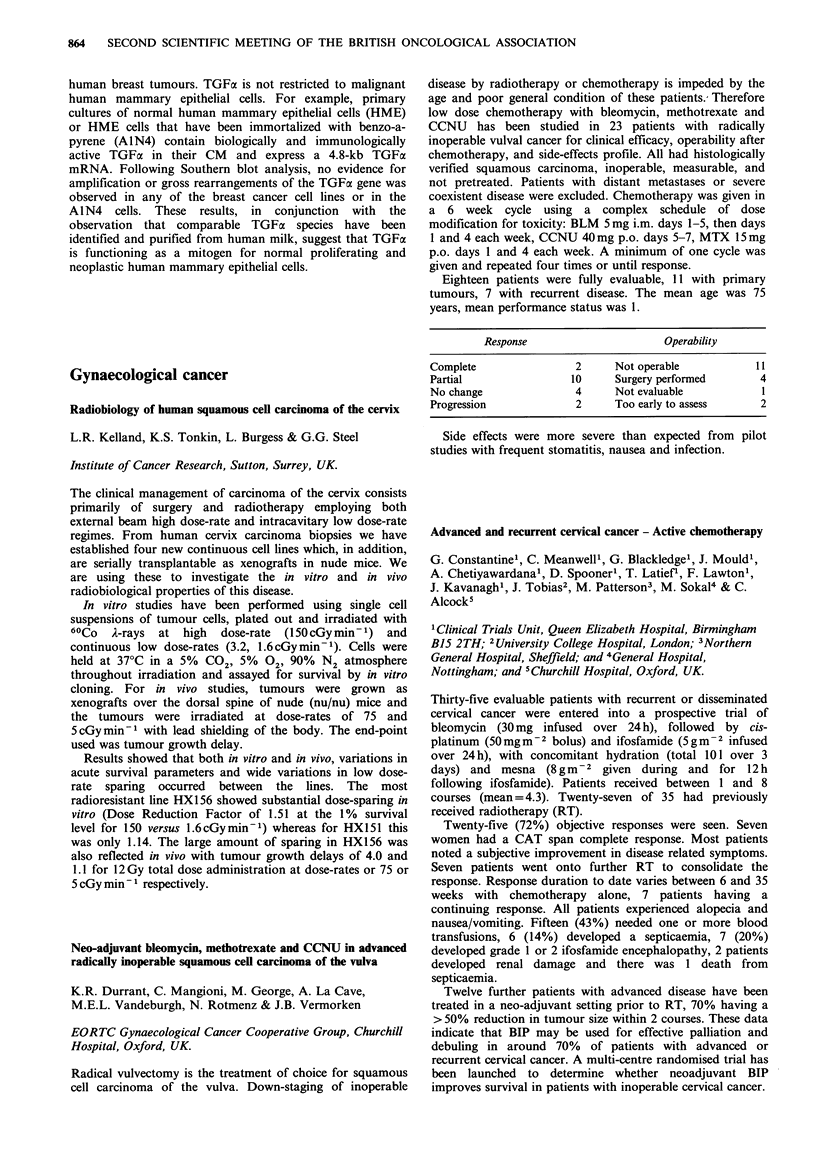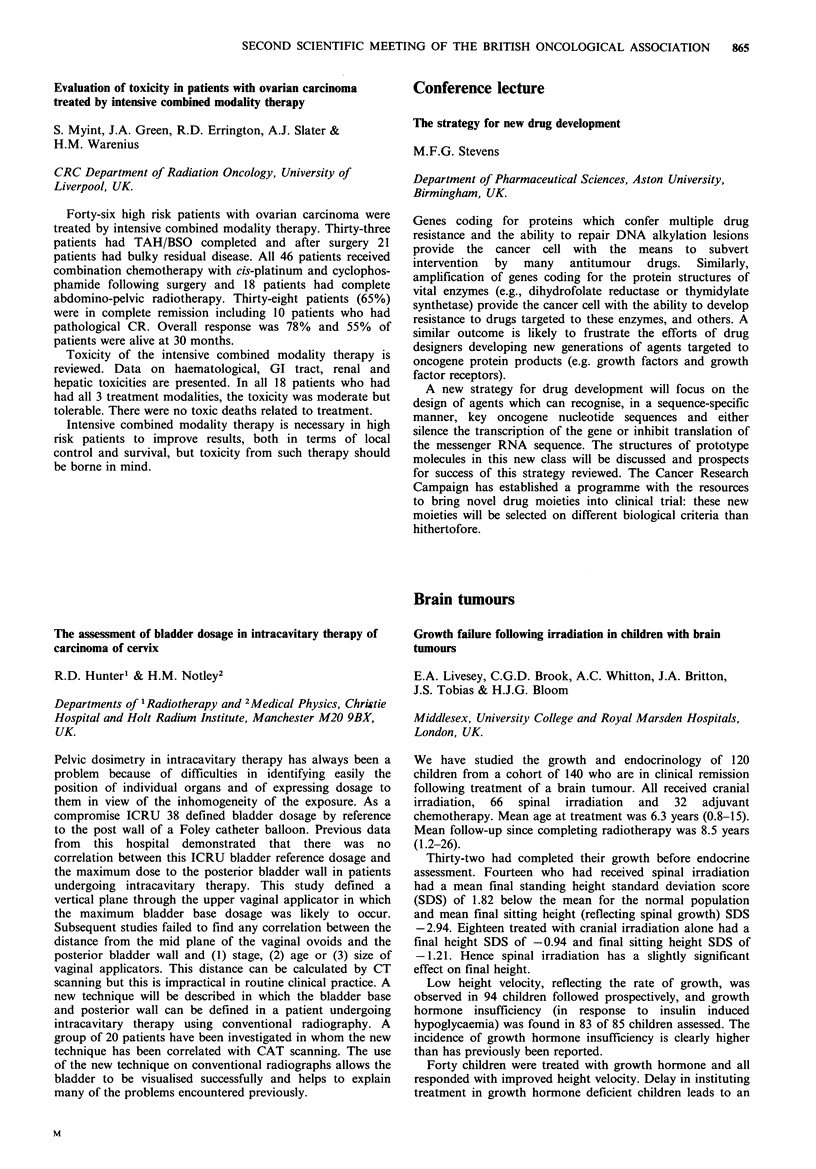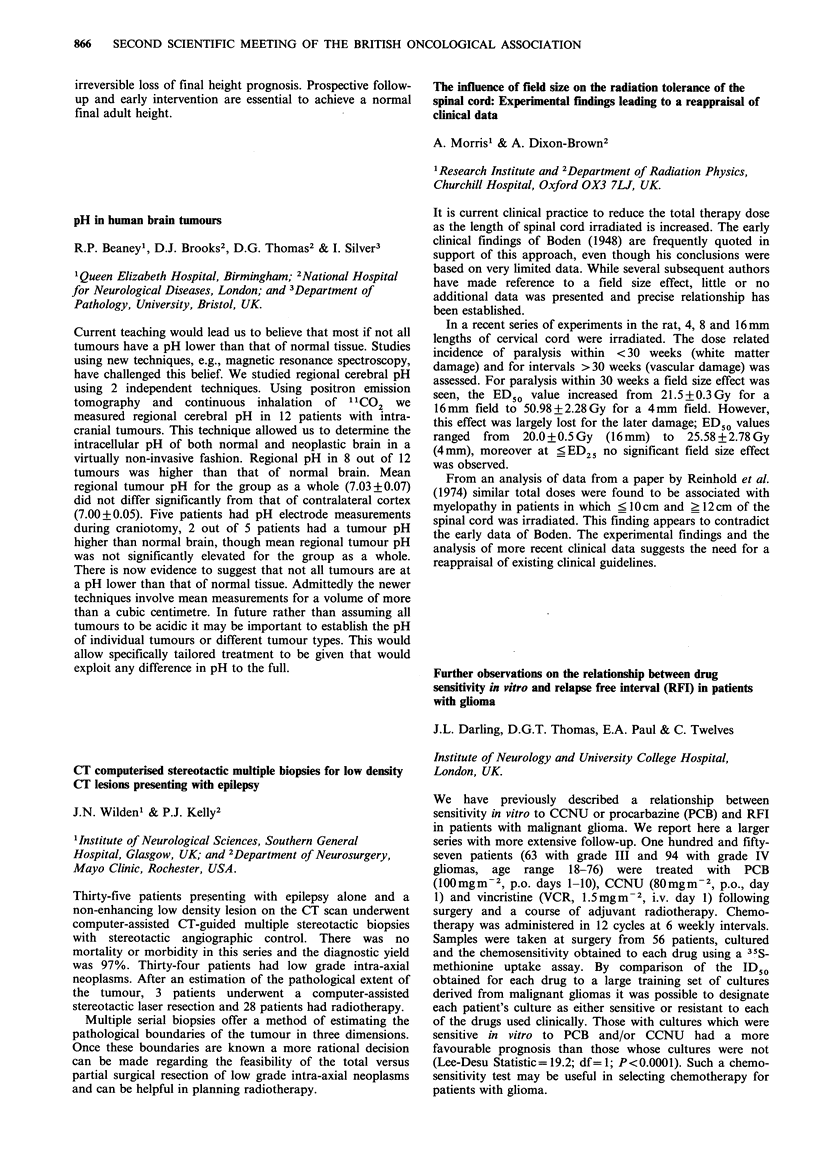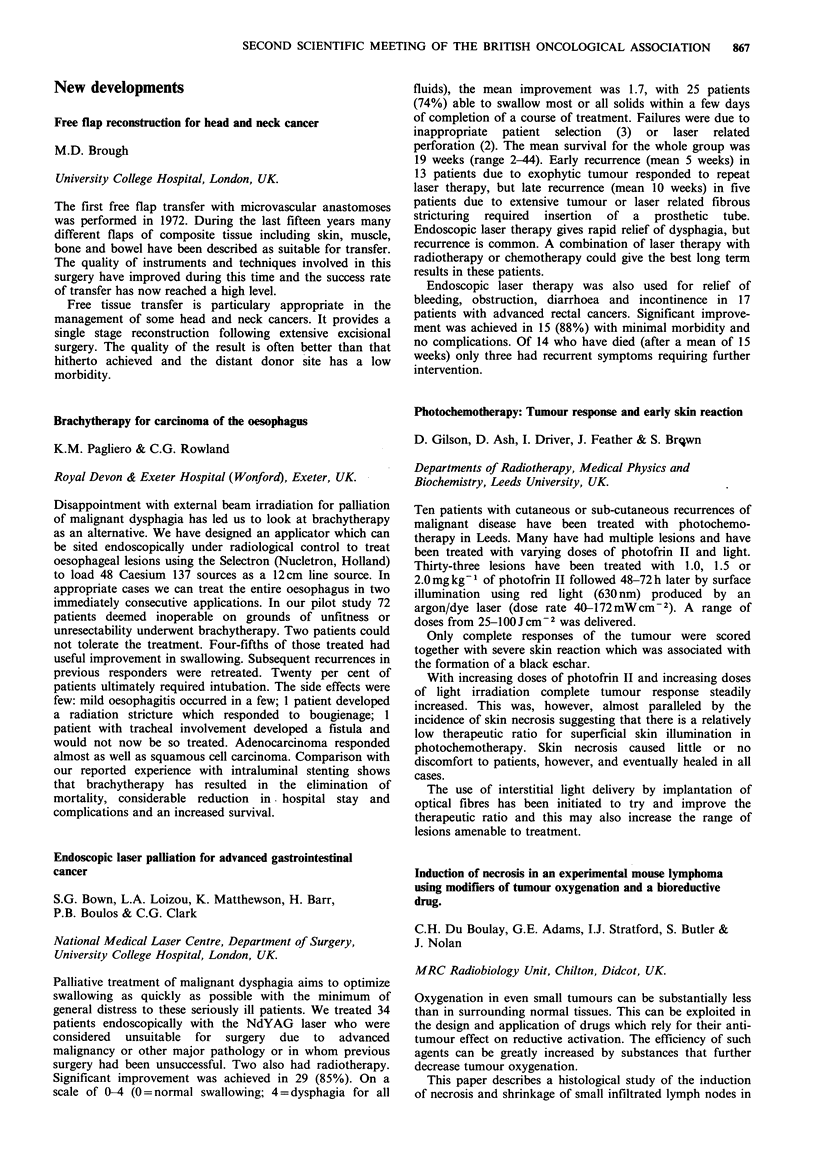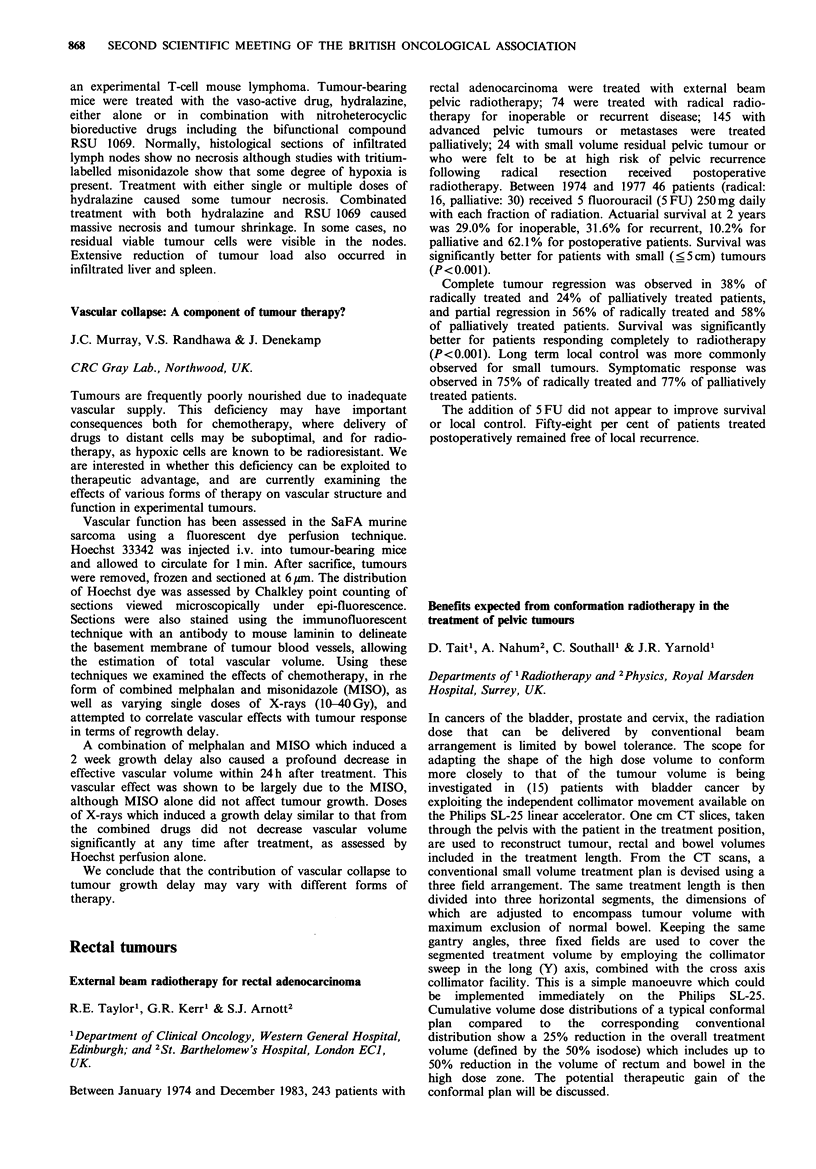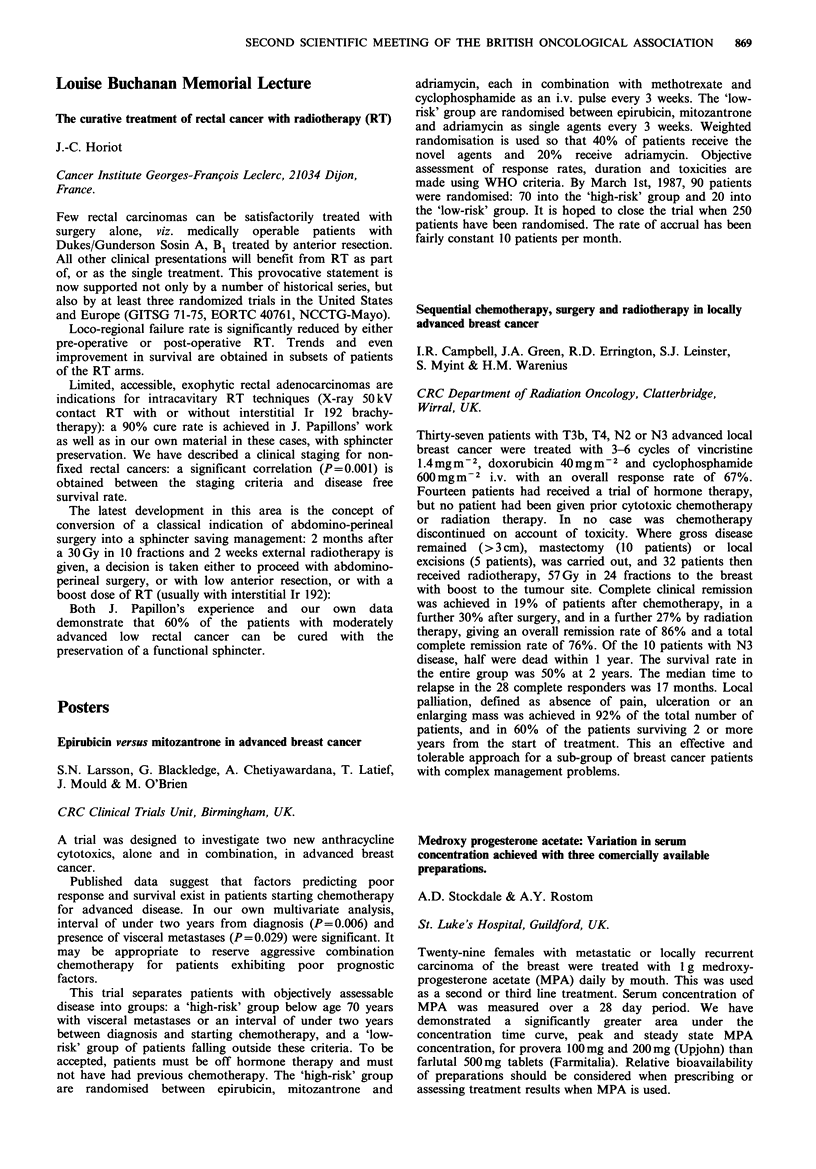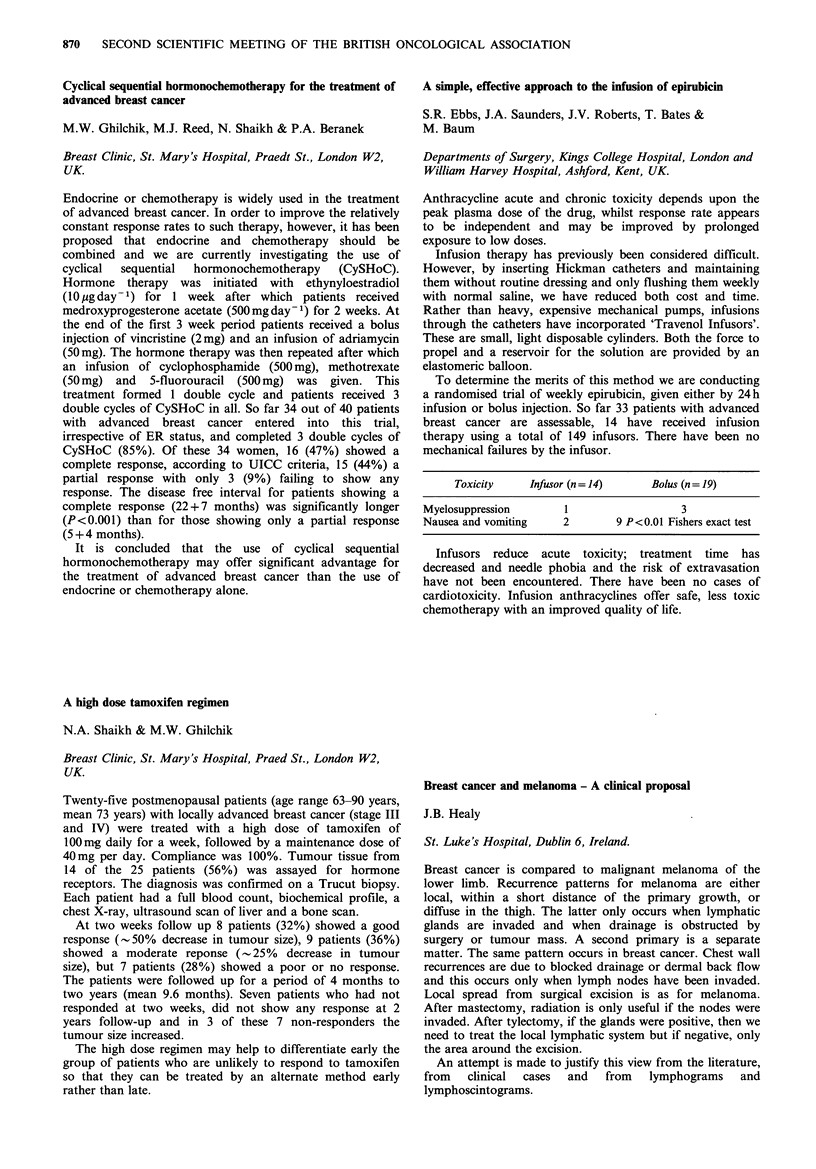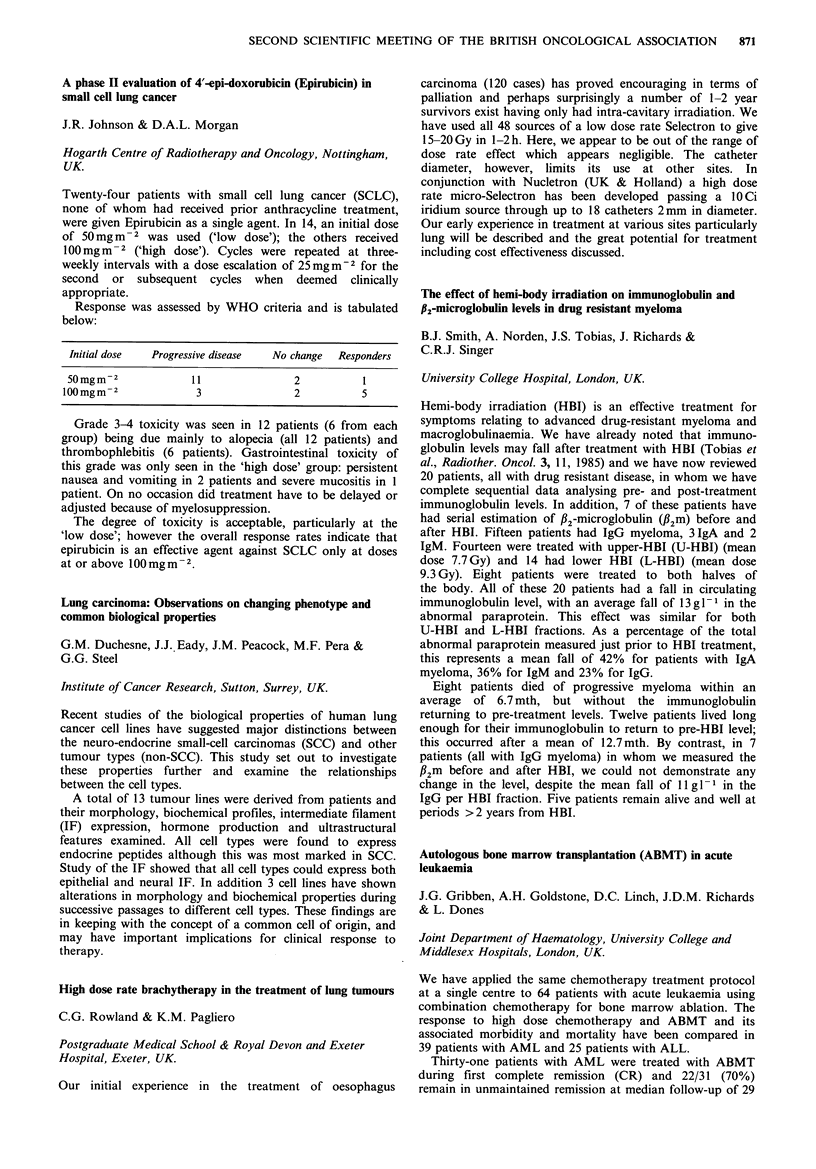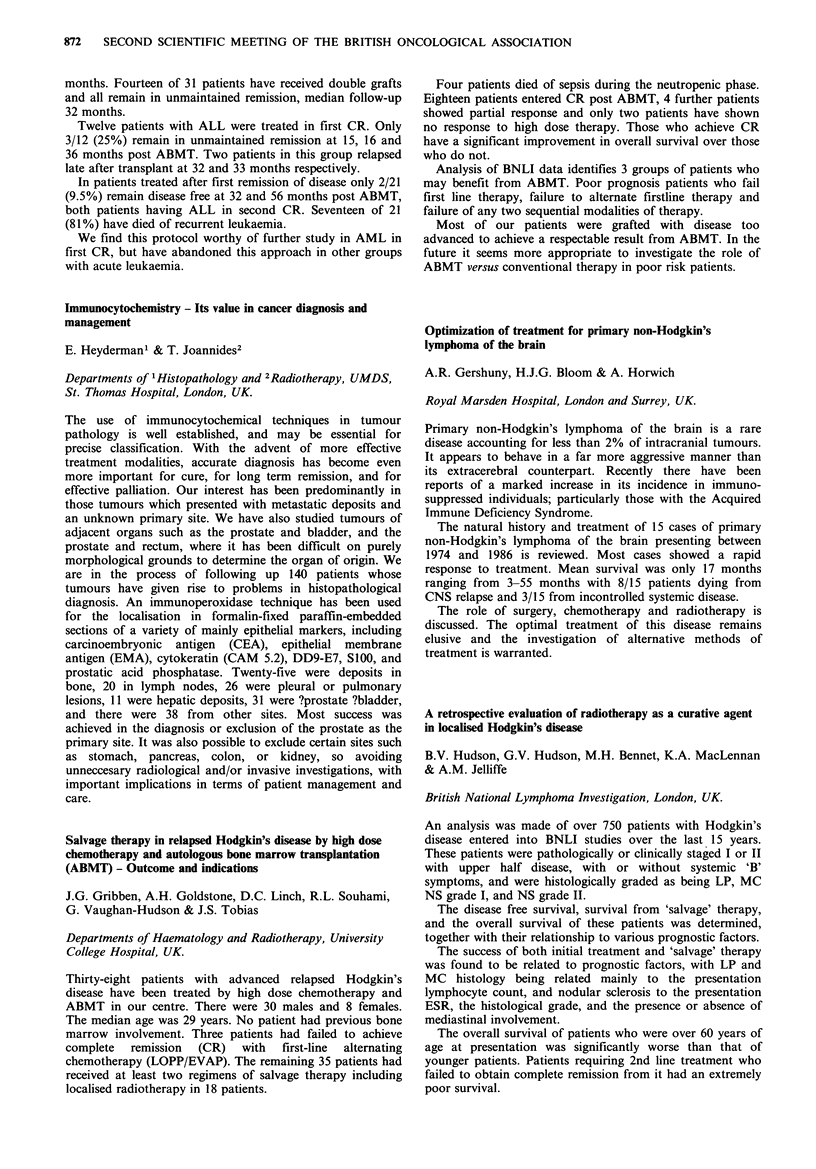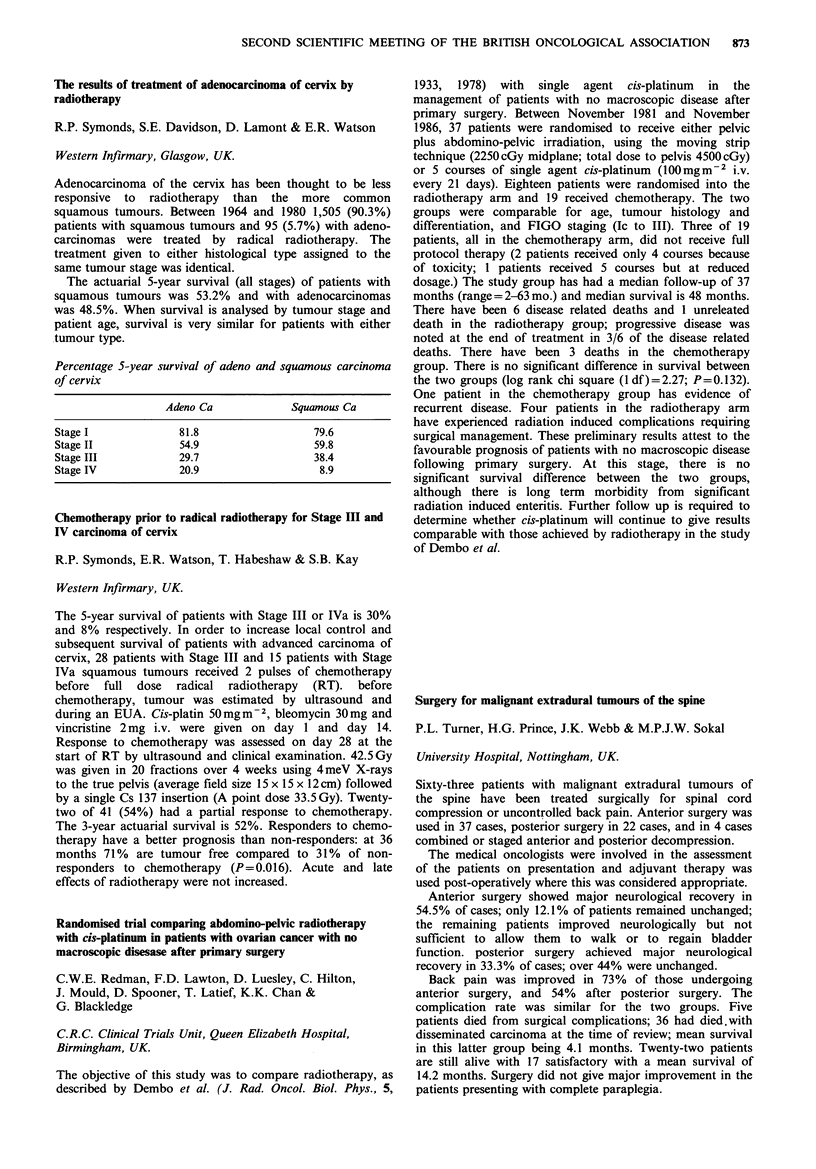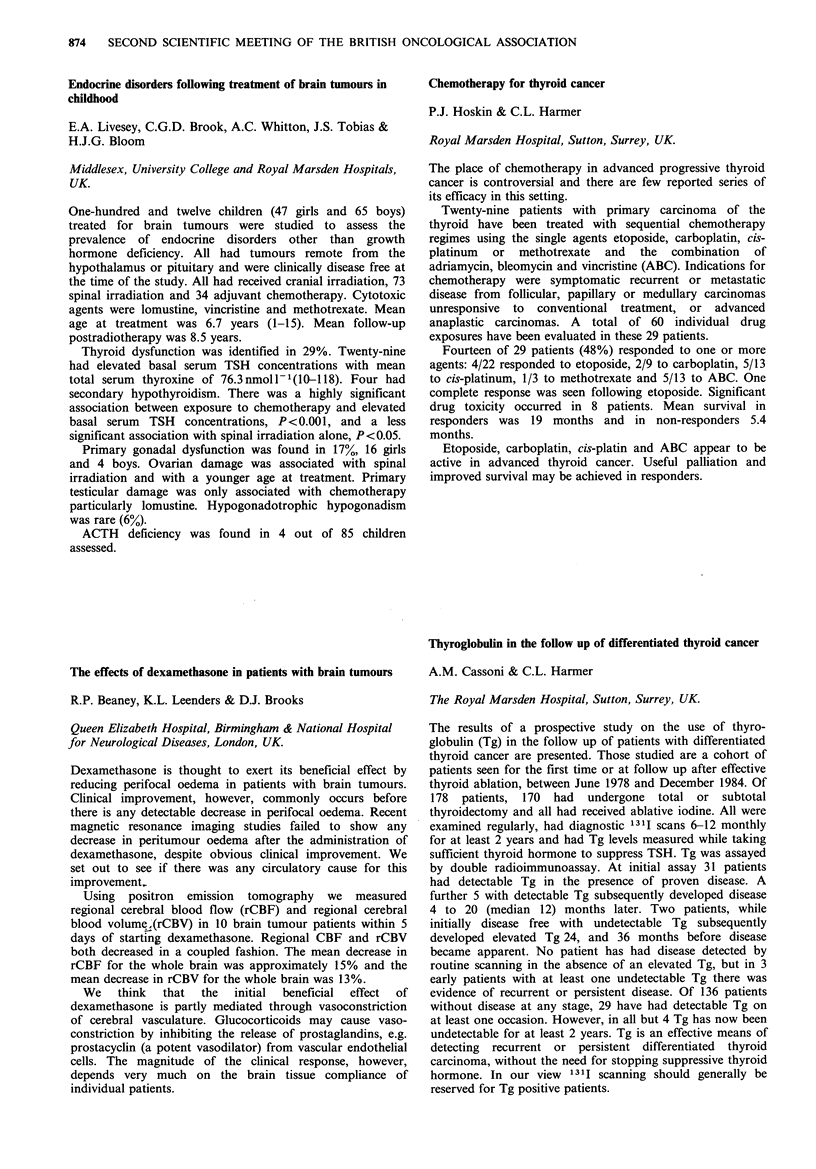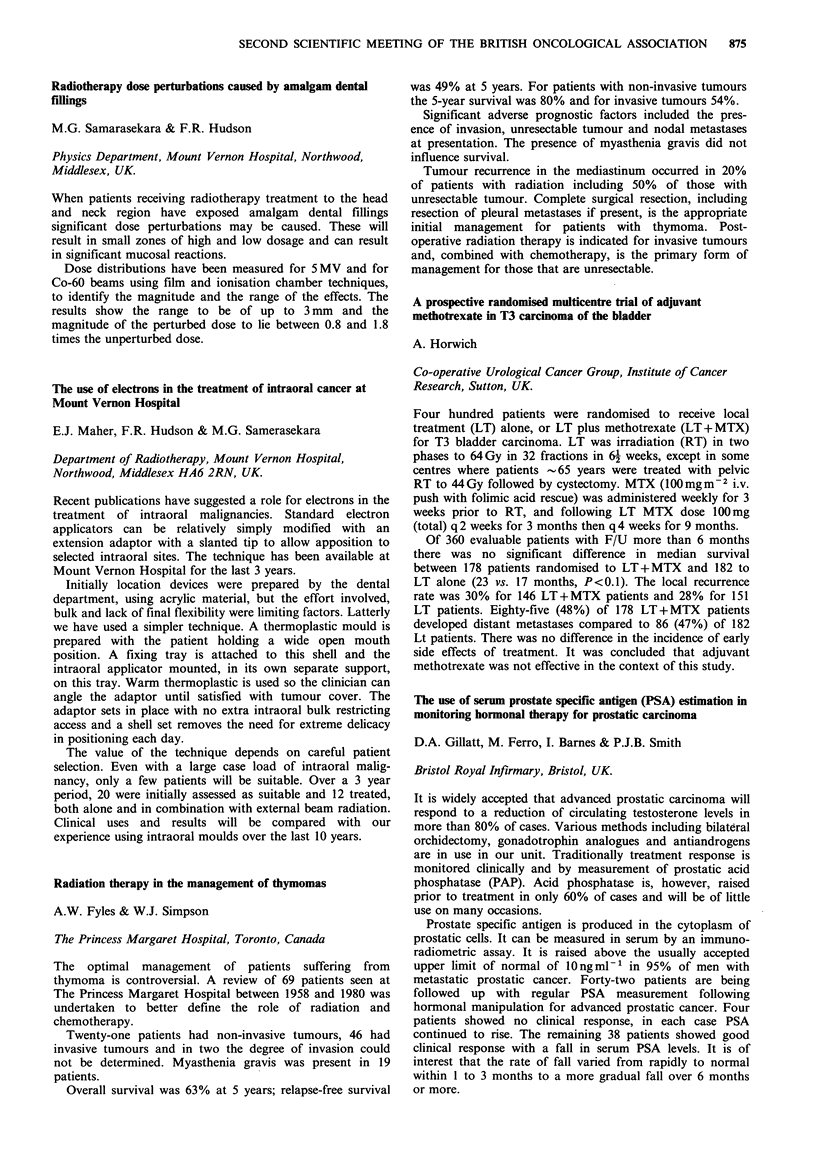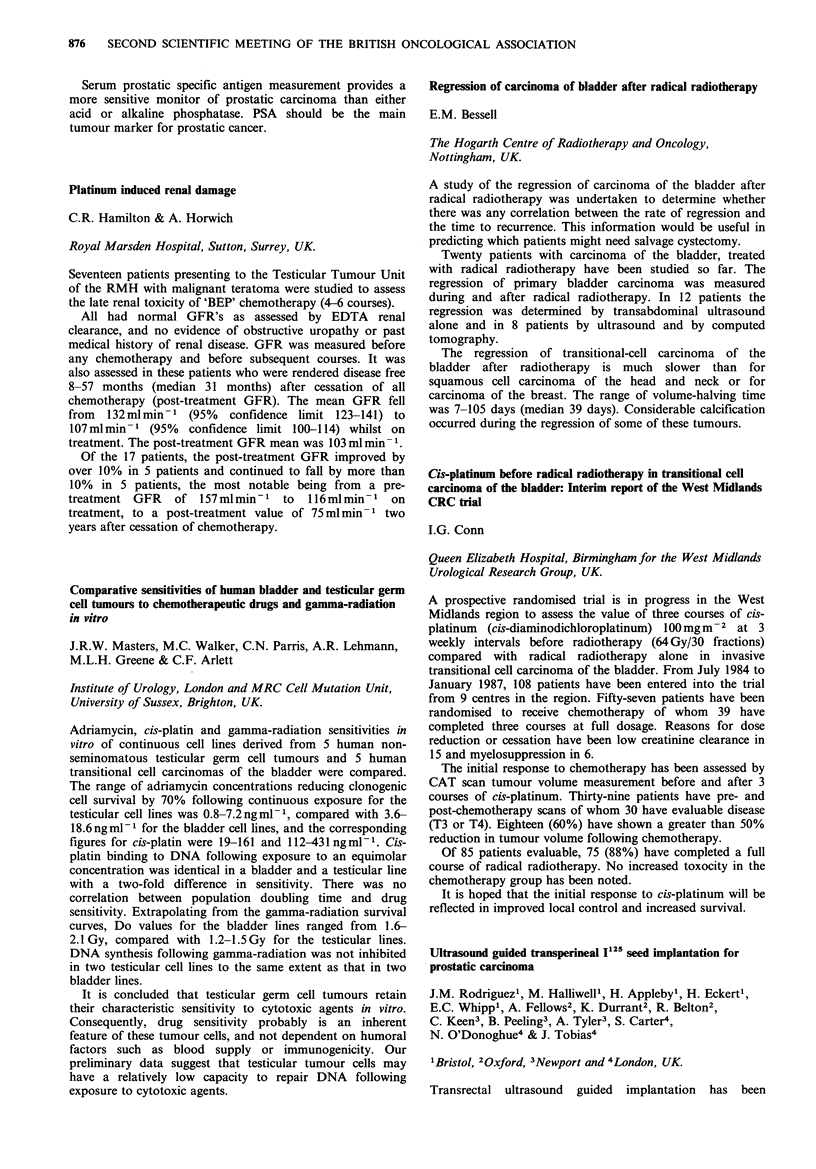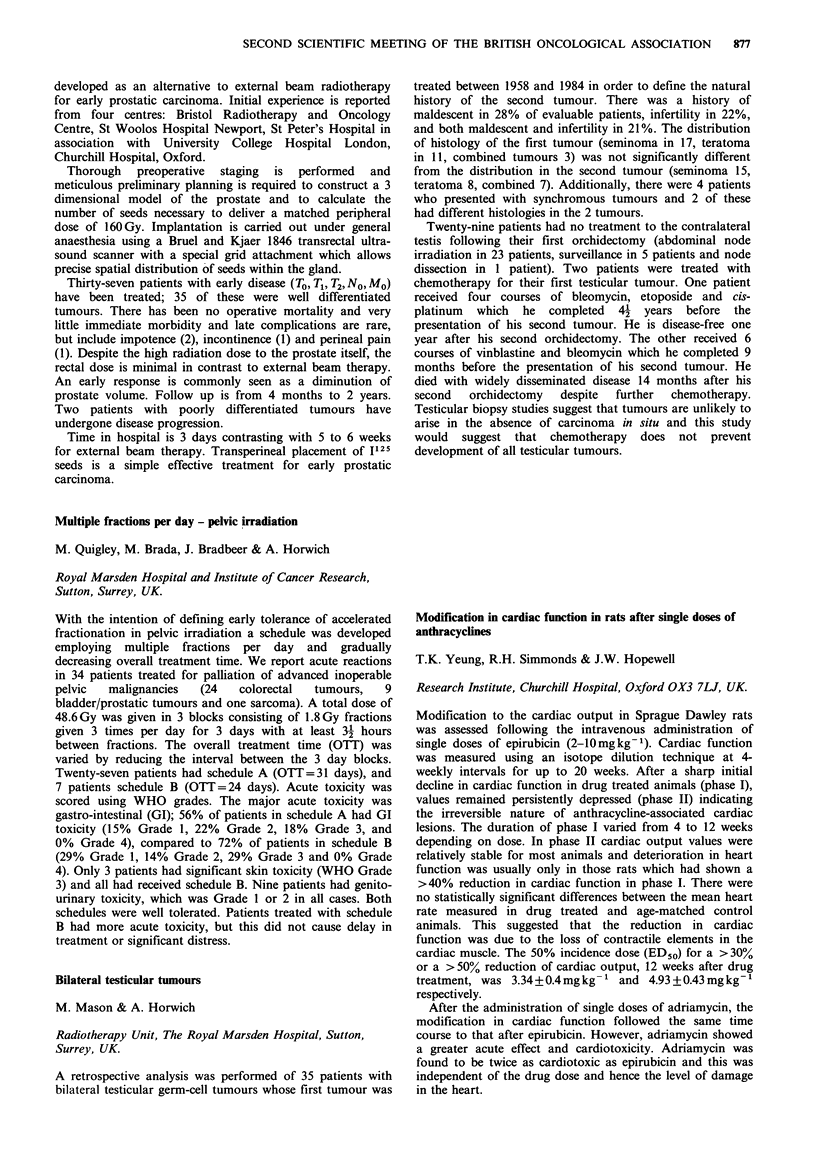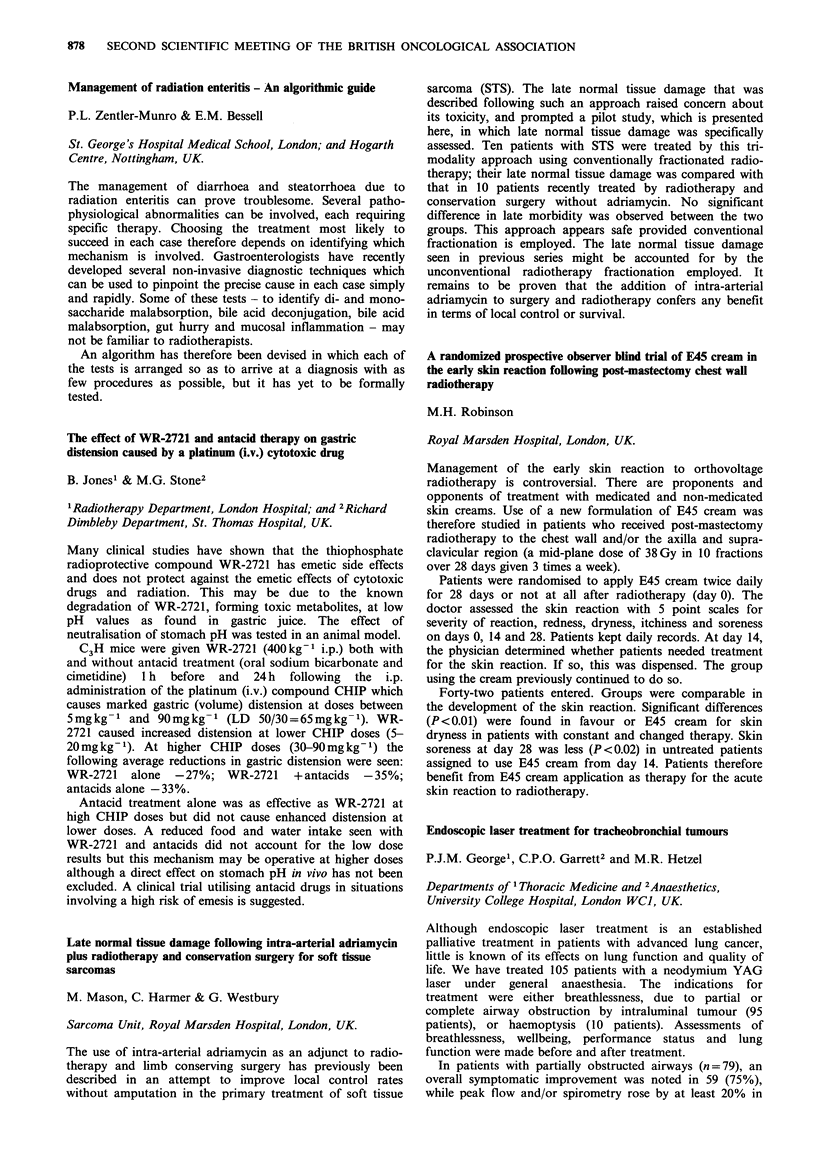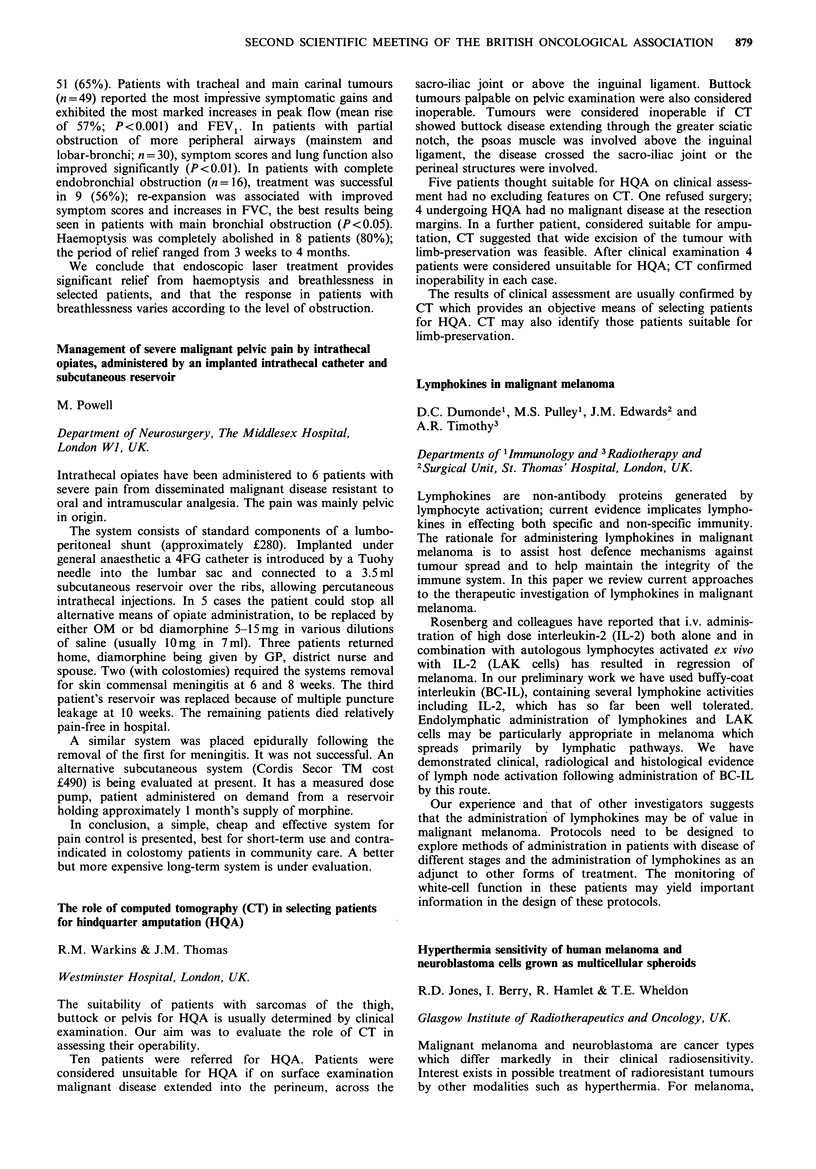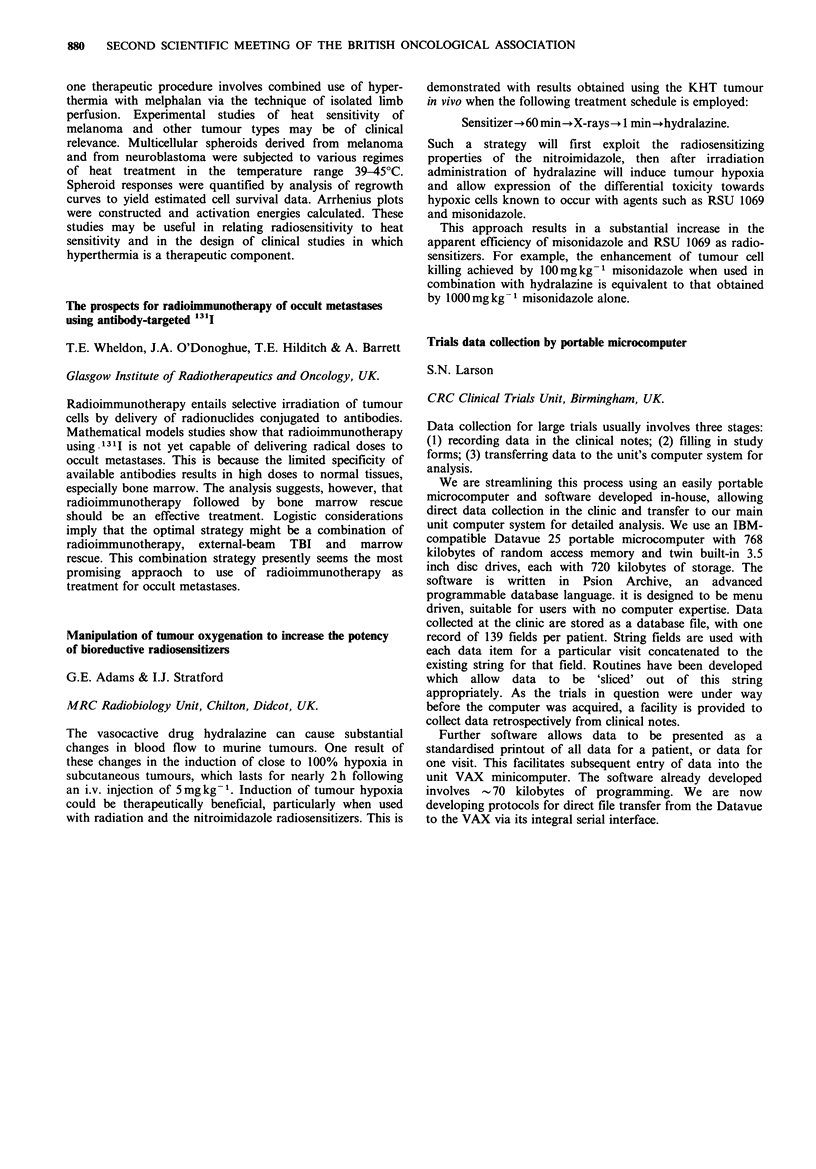# Second scientific meeting of the British Oncological Association. June 29-30, 1987, Oxford, UK. Abstracts.

**DOI:** 10.1038/bjc.1987.306

**Published:** 1987-12

**Authors:** 


Br. J. Cancer (1987), 56, 859–880 © The Macmillan Press Ltd., 1987

### Second Scientific Meeting of the British Oncological Association*

(Incorporating the Bob Champion Cancer Trust Lecture and the Louise Buchanan Memorial Lecture) June 29–30, 1987.

*Held at Wadham College, Oxford, UK.*

### Abstracts of papers†

### Breast cancer

### Prognostic value of cellular DNA content in the management of ductal carcinoma *in situ* of the female breast

R. Carpenter, J. Matthews, N. Gibbs, B. Thomas, P. Boulter & T. Cooke

*Charing Cross Hospital, Royal Surrey County Hospital, Guildford Breast Screening Centre and Liverpool Royal Infirmary, UK.*

A prognostic index for ductal carcinoma *in situ* (DCIS) of the female breast would allow a rational approach to management by selecting only the more aggressive lesions for radical local surgery. We have assessed cellular DNA content (ploidy) in screen detected, disaggregated DCIS by integrating microdensitometry after Feulgen staining in an attempt to identify phenotypically aggressive lesions.

Ploidy of DCIS ($n = 26$) associated with microinvasive carcinoma was compared with 12 cases of DCIS alone. Only 4 of 12 DCIS lesions was aneuploid compared with 23 of 26 DCIS lesions associated with microinvasive cancer ($P < 0.001$, $\chi^2 = 9.599$). In the screening programme to date at Guildford, there have been 5 local recurrences in 23 of 40 DCIS lesions treated by excision biopsy, all of the recurrent cases were originally aneuploid compared with only 6 of 18 cases not associated with recurrence ($P < 0.05$, $\chi^2 = 4.544$).

Aneuploidy is a frequent finding in DCIS which has progressed to the stage of micro invasion and has correctly predicted recurrence in women who have been treated by excision biopsy. Aneuploidy is of prognostic value and may allow selective management in DCIS of the female breast.

### Breast duct carcinoma *in situ*: Clinical data relating to its sensitivity

P. Price[1], A. McKinna[2], G. Walsh[2], B. Gusterson[3] & J.R. Yarnold[1]

*Departments of [1]Radiotherapy, [2]Surgery, [3]Pathology, Royal Marsden Hospital and Institute of Cancer Research, London, UK.*

Radiotherapy after lumpectomy in women with early stage breast cancer reduces the local recurrence rate from >30% at 5 years to <10%. The rationale of radiotherapy to the whole breast includes eradication of multifocal areas of duct carcinoma *in situ* (DCIS). However, the radiosensitivity of DCIS is unknown. A retrospective analysis has been performed in patients who have breast recurrence after lumpectomy ± radiotherapy for early stage breast cancer. The incidence of DCIS in relapsed patients has been investigated in terms of initial primary histology and whether radiotherapy had been given. Biopsy specimens have been independently reviewed from patients who relapsed in the breast after local excision for 1° invasive breast cancer (10 cases), 1° invasive breast cancer with DCIS (26 cases) and pure DCIS (7 cases). All patients had been treated by complete LE (1 cm clearance) and RT was given to the whole breast (usually = 60 Gy/30F/6 weeks).

DCIS was present in the recurrent tumour in more than one third of patients, regardless of whether radiotherapy had been given or not. On analysis, position, timing and pathology of the recurrent tumour suggests failure to eradicate original DCIS. These data suggest that high dose radiotherapy is relatively ineffective in eradicating DCIS in the vicinity of primary breast cancers and presumably elsewhere in the breast. These data question the use of radiotherapy to the whole breast after wide local excision for invasive breast cancer in the management of patients with pure DCIS treated by lumpectomy.

### A randomised cross-over trial of megestrol acetate *vs.* tamoxifen as initial treatment for post-menopausal women with advanced breast cancer

N. Stuart[1], C. Tyrell[2], D. Spooner[1], C. Keen[3], A. Taylor[4], J. Tarrant[4], G. Blackledge[1], D. Webster[5] & G. Rees[6]

*[1]Queen Elizabeth Hospital, Birmingham, [2]Plymouth General Hospital, [3]Velindre Hospital, Cardiff, [4]Stoke Mandeville Hospital, Aylesbury, [5]University Hospital of Wales, [6]Royal United Hospital, Bath, UK.*

Ninety-six peri- or post-menopausal patients (pts) have been entered into a randomised, cross-over trial comparing tamoxifen and megestrol acetate as first-line hormone treatment (HT) in advanced breast cancer. Interim results are presented without treatment codes being broken. No pt had received previous chemotherapy. All pts had disease measurable or evaluable for response. Median age was 64 (43–89). Forty-six of 88 pts on whom data is available presented with advanced disease and received study HT as primary treatment, 42/88 received study HT having relapsed a median of 48 months after primary treatment. Sites of dominant disease were: primary tumour, 37 pts; local recurrence, 6; nodal disease, 8; lung metastases, 11; bone metastases, 19; skin, 4; other, 2; no data (ND), 9.

Group A comprises 47 pts but 2 of these did not receive treatment. Thirty-two pts are evaluable for response, 1 (3%) achieved CR, 3 (9%) achieved PR, 16 (50%) had static disease for more than 3 months (SD) while in 12 (38%) disease progressed within 3 months (PD). Nineteen pts have completed treatment A and of these 14 have crossed over. Following cross-over 9 are evaluable for response: 6/9 PD, 3/9 SD. Group B comprises 45 pts but 2 of these did not receive treatment. Thirty-four pts are evaluable for response.

*Enquiries to the BOA Secretariat: J. Tobias, Department of Radiotherapy, University College Hospital, London WC1E 6AU.

†Reprints of these abstracts are not available – Ed.

3 (9%) achieved CR, 13 (38%) PR, 10 (29%) SD and 8 (24%) PD. Ten pts have completed treatment B and of these 8 have crossed-over. Following cross-over 6 are evaluable: 1 PR, 3 SD and 1 had PD. Toxicity has occurred in only a minority of pts in both groups. In group A 5/34 pts with follow-up data experienced toxicity during first treatment (fluid retention = 2 pts, glycosuria = 3 pts). In group B 6/34 pts experienced toxicity during first treatment (PV bleeding = 1pt, flushes = 3 pts, nausea = 2 pts). In all cases toxicity was mild (WHO grade 2 or less).

Interim analysis indicates that both treatments show broadly comparable response rates in post-menopausal women with advanced breast cancer. Both produce minimal toxicity. This study will continue until 200 pts have been randomised. Final end-points will include initial and cross-over response rates, toxicity, time to progression and survival.

### Affecting the natural history of breast cancer

J.R. Harris & Samuel Hellman[2]

[1]*Joint Center for Radiation Therapy, Harvard Medical School, Boston and* [2]*Memorial Sloan–Kettering Cancer Center, New York, USA.*

The many changes in breast cancer management offer an opportunity to review the hypotheses on which these are based and the evidence that such treatments affect ultimate survival. Traditional treatment has been based on the notion that the disease, in many patients, is an orderly one starting with local disease and then spreading to the regional lymph nodes and finally distantly. The alternative hypothesis is that if the disease is to demonstrate metastatic potential, it metastasizes before clinical presentation and thus local and regional treatment are of little or no value. Current evidence seems to argue against the latter hypothesis being regularly the case since breast cancer mortality can be reduced by as much as 30% using mammographic screening. Local control appears to influence the proportion of distant metastases seen in randomized control clinical trials. Even in patients with positive nodes improved local control is associated with improved survival. Systemic therapies are also available for the treatment of occult distant metastases. While evaluation is limited by the restricted follow-up time, it is clear that survival curves are altered and that survival is prolonged. What is not clear is whether the proportion of patients cured of the disease has changed.

### Radiation induced brachial plexus damage and time-dose fractionation – A clinical and CT study

S. Powell, J. Cooke & C. Parsons

*Royal Marsden Hospital, London, UK.*

The radiation tolerance of the brachial plexus was investigated in 459 patients with breast cancer treated post-operatively. The effects of two radiation schedules were compared: 60 Gy in 30 fractions or 51 Gy in 15 fractions, both over 6 weeks. Patient follow-up was 30–58 months after radiotherapy. The incidence of neurological damage to the brachial plexus was 2.7% with large fraction size (3.4 Gy) and <1% with small fraction size (2 Gy).

Computed tomography (CT) of the root of neck and axilla, using bolus i.v. contrast to improve visualisation of the brachial plexus, was performed in 42 patients (44 CT scans). A pattern of increased soft-tissue density (ISD) surrounding the brachial plexus was interpreted as a post radiation effect, distinguishable from recurrent disease. Ninety-six per cent (27/28) of patients with a clinically detectable brachial plexus lesion had ISD, while only 50% (8/16) of patients with no neurological deficit had ISD. The pattern of ISD was not related to nodal disease status, type of axillary surgery or technique of radiotherapy. The degree of ISD (graded mild, moderate or severe) appears to be associated with large fraction size. Fifty-four per cent (17/31) of patients receiving large dose per fraction had moderate or severe ISD, compared with 23% (3/13) of patients treated with small dose per fraction.

It is concluded that CT can usefully assess the degree of post radiation effect, and that the radiation tolerance of the brachial plexus is largely dependent on fraction size. The relationship between total dose and fraction size can be calculated.

### Elucidating the action of the antioestrogens? Response to toremifene (Fc-1157a) therapy in tamoxifen failed patients

S.R. Ebbs, J.V. Roberts, A.J. Wilson & M. Baum

*Department of Surgery, Kings College Hospital, London, UK.*

The clinical activity of the triphenylethylene group of drugs does not demonstrate an absolute correlation with their anti-oestrogenic activity or the oestrogen receptor content of the tumour.

Toremifene (Fc-1157a) is a new triphenylethylene anti-oestrogen which at low concentrations produces effects comparable with tamoxifen. At higher dose toremifene exerts anti-tumour effects, some of which are different from those of tamoxifen and are directed against oestrogen receptor negative breast tumours and tumours of mesenchymal origin. The exact mechanism of these effects is unknown.

To determine whether this effect seen in experimental cell lines and animal models is reproducible in man we have used toremifene as a second line therapy in patients who have relapsed whilst receiving tamoxifen.

In 9 patients treated for over 3 months toremifene produced a partial response in 3, (33%) and no change in 3, (33%) using UICC criteria.

A single patient with multiple desmoid tumours who relapsed after responding to tamoxifen also responded dramatically to toremifene.

As this is a group of patients who had relapsed whilst receiving tamoxifen, it may be that, as in cell culture and animal experiments, we are witnessing an alternative pathway of action perhaps mediated by effects on mesenchymal cells.

### Conference Lecture

### Early breast cancer trialists collaboration

R. Peto

*Clinical Trial Service Unit, Radcliffe Infirmary, Oxford, UK.*

Taken in isolation, most clinical trials in early breast cancer would not be capable of determining reliably whether a particular adjuvant treatment had (a) no material effect on survival, or (b) a moderate yet humanly worthwhile effect (e.g. reducing 5-year mortality from 1/3 down to 1/4). An overview of all available randomised trials might, however, be accurate enough for this purpose, and a large number of trialists have therefore chosen to collaborate in such an overview. Some of the main results from this collaboration will be presented on their behalf. Among women aged over 50, 2 years of tamoxifen reduced 5-year mortality from 33% to ~25%, an effect that was overwhelmingly statistically significant. Among women under 50, however, the number of women available for review was smaller, and no direct evidence of an effect of tamoxifen on mortality existed, either because it has little effect on mortality or because the results were distorted by the play of chance. For polychemotherapy, the opposite pattern was apparent: it produced a highly significant mortality reduction among women under the age of 50, but a smaller and less clearly significant effect among older women. For both agents, the *proportional* changes in outcome appeared similar for women with no, few or many axillary nodes. The effects on long-term survival after 5 years are not yet known.

### Lung cancer

### Radiotherapy employing 3 fractions each day over a continuous period of 12 days

M.I. Saunders & S. Dische

*Marie Curie Research Wing, Mount Vernon Hospital, Northwood, Middlesex, UK.*

Studies in the cell kinetics of human tumours have given support to the use of accelerated fractionation to overcome regrowth of tumours between fractions. Any pause in treatment as may occur with a split-course schedule, or even for the week-end, is likely to negate the benefit to be obtained. We have administered 36 treatments over a continuous period of 12 days with a 6 hour gap between each of the three treatments given each day. Seventy-four patients with intra-thoracic and head and neck tumours have now been included in this study. Acute reactions have been tolerable and it was found possible to increase the minimum tumour dose from a total of 50.4 to 54.0 Gy. Immediate tumour responses have been promising. Twelve (46%) of 26 assessable patients with carcinoma of the bronchus have shown complete radiological regression and this can be compared with 9 (16%) of 62 similar patients included in a previous study. Twenty-one patients with advanced head and neck tumours have shown a rapid response in the primary site with complete regression and healing occurring in all except one case. The method has proved to be a practical one and the promising tumour responses, together with the expectation of a low incidence of late changes, encourages further exploration of this regime.

### Feasibility study of alternating radio-chemotherapy using multiple fractions per day in patients with small cell lung cancer (SCLC)

D. Parton[1], J.R. Yarnold[1] & I.E. Smith[2]

*Departments of [1]Radiotherapy and [2]Medicine, Royal Marsden Hospital, Surrey, UK.*

Alternating courses of cytotoxic chemotherapy and hyper-fractionated radiotherapy may improve the therapeutic ratio in LDSCLC. Fourteen patients were entered in to a feasibility study of hyperfractionated radiotherapy intercalated between courses of induction chemotherapy. WHO performance status at presentation was 0 or 1 in all patients. Median age at presentation was 61 years, range 50–69. Five courses of JM8 (400 mg m$^{-2}$ day 1), Ifosfamide (5 g m$^{-1}$ day 1), VP16 (100 mg m$^{-2}$ days 1–3) were given at 28 day intervals. Five days after the first and second courses of chemotherapy a 5 day course of radiotherapy was given to the primary tumour and mediastinum by AP opposed fields using 5 Mev X-rays. Each course of radiotherapy delivered 15 Gy tumour dose in 15 fractions over 5 consecutive days with a 3 h gap between fractions. During the period of intercalated radio-chemotherapy, performance status remained unchanged or improved in 9/14 patients and deteriorated in 5 patients including 1 death. Six of 27 (22%) courses of radiotherapy were associated with oesophagitis (all WHO grade III); onset was the sixth day after start of radiotherapy with resolution within 8 days. Eight of 14 patients have been followed up for a minimum of 2 months following completion of radiotherapy and 4 have radiological pneumonitis (1/4 has clinical pneumonitis). In conclusion, the incidence and severity of oesophagitis and pneumonitis are higher than expected and may reflect enhancement by high dose cytotoxic agents, especially ifosfamide. Tumour response rates are high and the protocol continues but with limited scope for escalating the radiation dose.

### Chemotherapy for cerebral metastases in small cell carcinoma of the lung (SCCL)

C.J. Twelves[1], J.S. Tobias[2], P.G. Harper[1], C.M. Ash[2], B. Mantell[3], R.L. Souhami[2], S.G. Spiro[4] & D.M. Geddes[5]

*[1]Guy's, [2]University College Hospital, [3]The London Hospital, [4]Brompton Hospital, [5]London Chest Hospital, London, UK.*

Within a chemotherapy trial for SCCL, patients with cerebral metastases were identified as a special sub-group. By withholding cranial irradiation the aim was to evaluate the effect of chemotherapy as initial treatment for cerebral metastases. Responses were objectively assessed by a series of CT brain scans.

Twenty-five patients (4.1%) had CT proven cerebral metastases at presentation, but 5 underwent cranial irradiation, and 2 craniotomy prior to chemotherapy. The remaining 18 patients were treated with cyclophosphamide 1 g m$^{-2}$ i.v. day 1, vincristine 2 mg i.v. day 1 and etoposide 100 mg t.d.s. p.o. days 1–3, q3w. Steroids were given where clinically indicated at the lowest possible dose.

Nine of 14 patients who had a repeat CT scan achieved a CR or PR. One of the 4 clinically assessable patients also improved, giving an overall response rate of 54%. Radiological responses were rapid, sustained, accompanied by clinical improvement and included 7 patients who had not received steroids.

Chemotherapy has previously been discounted as treatment for cranial metastases because of the assumption that the blood brain barrier (BBB) protects them from systemic chemotherapy. In fact metastases develop their own tumour circulation which probably has no BBB. In SCCL cerebral metastases respond to chemotherapy which may be considered for first-line palliative treatment, having several advantages over radiotherapy.

### Treatment duration in small cell lung cancer (SCLC). A randomised comparison of 4 *versus* 8 courses of initial chemotherapy

J.S. Tobias[1], R.L. Souhami[1], C.M. Ash[1], S.G. Spiro[2], D.M. Geddes[3], P.G. Harper[4], H.M. Earl[1] & H. Quinn[1]

*[1]University College Hospital, [2]Brompton Hospital, [3]London Chest Hospital and [4]Guy's Hospital, London, UK.*

Six hundred and sixteen patients with SCLC were entered into a randomised trial comparing different treatment durations and the value of chemotherapy on relapse. Patients were staged by isotope bone scan and liver ultrasound, and stratified according to stage (limited or extensive). Patients were then randomised to receive either 4 or 8 courses of chemotherapy (cyclophosphamide 1 g m$^{-2}$ day 1, vincristine 2 mg day 1, etoposide 100 mg t.d.s. days 1–3) 3 weekly. At presentation patients were also randomised for treatment at relapse, either to receive further chemotherapy (doxorubicin $50\,mg\,m^{-2}$, and methotrexate $50\,mg\,m^{-2}$, every 3 weeks) or symptomatic treatment alone.

Response rates to short (S) and long (L) initial chemotherapy were similar (S=61%, L=63%), as were response rates to relapse chemotherapy (S=25%, L=18%). Overall median survival (MS) from course one was analysed by intention to treat, and patients randomised to 8 courses of initial chemotherapy had a slightly longer MS than those randomised to 4 courses (MS, 39 *vs.* 32 weeks, $P=0.085$). Progression free interval (PFI) after initial chemotherapy was longer in patients receiving 8 rather than 4 courses (Median, 31 *vs.* 23 weeks, $P=0.0002$). Survival from relapse was longer for patients receiving relapse chemotherapy than for those receiving symptomatic treatment alone (MS, 17 *vs.* 12 weeks, $P=0.0004$). Patients who received short course chemotherapy, and no further chemotherapy at relapse, clearly survived for a significantly shorter time than the other three treatment groups.

### Long-term follow-up of 72 patients given prophylactic cranial irradiation for small-cell lung cancer

J.R. Johnson & D.A.L. Morgan

*Hogarth Centre of Radiotherapy and Oncology, Nottingham, UK.*

The role of prophylactic cranial irradiation (PCI) in the combined-modality management of small-cell lung cancer (SCLC) remains unsettled. We have analysed the long-term results of such treatment as employed in a standard fashion at a single institution over a 5-year period.

From 1978 to 1983, 72 patients with SCLC received PCI, of whom 58 had limited disease and the irradiation was part of induction treatment; 14 had extensive disease and were treated after showing a good response to chemotherapy.

The technique employed utilised opposed portals, with no compensators, delivering a mid-plane dose of 30 Gy in 10 fractions from either a Cobalt-60 Unit or a 6 MV linear accelerator. A standard chemotherapy regimen comprising vincristine, adriamycin and cyclophosphamide was used.

Fourteen of the patients subsequently developed brain metastases, 2 developed severe non-metastatic neurological impairment, and 4 died of neurological disease of uncertain aetiology (CT scanning was not available in Nottingham at that time). Three patients are alive, well and disease-free.

PCI entails considerable morbidity, and is of unproven value: its use should be restricted to prospective evaluation in controlled clinical trials.

### The dose-rate effect and recovery in human tumour cells

G.G. Steel, J.M. Deacon, G.M. Duchesne, A. Horwich, L.R. Kelland & J.H. Peacock

*Radiotherapy Research Unit, Institute of Cancer Research, Sutton, Surrey, UK.*

The radiation response of 12 cell lines derived from a variety of human tumours has been investigated over the dose-rate range from 150 to $1.6\,cGy\,min^{-1}$. As the dose-rate was lowered, the amount of sparing varied widely; in two cell lines it was zero, in the other cell lines the dose required for $10^{-2}$ survival ranged up to twice the value at high dose-rate. Low dose-rate irradiation discriminates better than high dose-rate between tumour cell lines of differing radiosensitivity. The data are equally well fitted by two mathematical models of the dose-rate effect: the LPL model of Curtis and the Incomplete Repair model of Thames. Analysis by the LPL model leads to the conclusion that the theoretical radiosensitivity in the total absence of repair was rather similar among the 7 cell lines on which this analysis was possible. What differs among these cell lines is the extent of repair and/or the probability of direct infliction of a non-repairable lesion. Recovery from radiation damage was also examined by split-dose experiments in a total of 17 human tumour cell lines. Half-time values ranged from 0.36 to 2.3 h and there was a systematic tendency for split-dose halving times to be longer than those derived from analysis of the dose-rate effect. This could imply that cellular recovery is a two-component process, low-dose rate sparing being dominated by the faster component.

### Lymphomas and leukaemia

### Stage III Hodgkin's disease – Long term results

M. Brada, J. Nicholls, S. Ashley, M. Coleman, M.J. Peckham & A. Horwich

*Institute of Cancer Research and Royal Marsden Hospital, Sutton, Surrey, UK.*

We performed a retrospective analysis of 215 patients with clinical (CS) and pathological Stage (PS) III Hodgkin's disease (HD) treated at the Royal Marsden Hospital between 1963 and 1985 (median follow-up of patients alive – 9 years; range 1–21 years). Eighty-four patients had PSIII (initial CSI & II) and 131 patients CSIII (53 laparotomised) histologically confirmed HD. All had infra-diaphragmatic assessment by lymphography and/or CT scan. The following prognostic factors were analysed: age, sex, histology, presenting level of haemoglobin and ESR, systemic symptoms, sites, extent and bulk of disease. In addition we assessed the influence of initial treatment modality.

The actuarial 5 and 10 year survival was 77% and 65% respectively, with 56% and 49% 5 and 10 year disease-free survival. Although many factors affected the disease-free survival, the only major prognostic indicator for survival was age.

Ninety-one patients were initially treated with combined chemotherapy and radiotherapy (CMT). Their survival was significantly better when compared to patients treated with radiotherapy (73 patients) or chemotherapy (51 patients) alone. When corrected for age, patients under 40 years treated with CMT demonstrated an improved survival, but this did not reach statistical significance. The role for CMT in Stage III HD should be tested in a prospective randomized trial.

### Bone marrow transplantation in lymphoblastic lymphoma in remission – Autologous *versus* allogenic

J.G. Gribben, A.H. Goldstone, L. Dones & P. Ernst

*The EBMT Lymphoma Group.*

Seventy-four patients with lymphoblastic lymphoma have been treated by bone marrow transplantation in complete remission of disease, 27 by allogenic transplant and 47 by autograft. All of the allografted patients were conditioned using a total body irradiation (TBI) containing regimen, but TBI was used in only 13/47 in the autograft group.

At the time of analysis 49 patients were alive, 18/27 (67%) in the allograft group and 31/47 (66%) in the autograft group. Six patients died during the procedure, 3 (11%) of the allografts and 3 (6%) in the autograft group. Nine (33%) of those allografted developed GVHD assessed as Grade II or greater.

Relapse of disease was the principal cause of death in both groups. Patients transplanted in first CR had a disease free survival advantage over those transplanted in subsequent CR.

The source of marrow, whether autologous or allogenic, was found not to influence overall survival or probability of relapse.

### Central nervous system involvement in patients receiving autologous bone marrow transplantation for non-Hodgkin's lymphoma

J.G. Gribben, A.H. Goldstone, L. Dones

*Department of Haematology, University College Hospital, London, UK.*

Of 309 patients reported to the European Bone Marrow Transplant Group (EBMT) with non-Hodgkin's lymphoma who have been treated by autologous bone marrow transplantation (ABMT), 24 (7.8%) had CNS involvement. There were 21 males and 3 females. Twelve were children aged less than 15 years and 12 were aged 16–58 (median 43) years. Three had intermediate grade histology, 6 had high grade lymphoblastic, 6 had other high grade and 11 had Burkitt's lymphoma.

Fourteen had CNS involvement at the time of diagnosis and of these 3 still had CNS involvement at the time of ABMT. Ten further patients had CNS involvement at relapse so that 13 patients had CNS disease at the time of ABMT.

Fifteen of 24 (62.5%) achieved or maintained complete remission (CR) post ABMT. Of these, 9 patients were in CR at the time of ABMT and 2 (22%) have subsequently had CNS relapse. The remaining 7 patients remain alive and disease-free at 3–28 months post ABMT. Of 13 patients who had CNS involvement at the time of ABMT only 4 (30%) remain alive at 23–68 months post ABMT.

The overall survival of the 24 patients is not different from that of the total NHL group ($P=0.67$). Those patients who had CNS disease at the time of ABMT have poor survival.

### The effect of immunohistological diagnosis on the clinical management and prognosis of malignant tumours of uncertain origin

M.H. Robinson[1], C.J. Alcock[2] & D.Y. Mason[3]

*[1]Royal Marsden Hospital, London, [2]Churchill Hospital, Oxford, and [3]John Radcliffe Hospital, Oxford, UK.*

The value of a panel of monoclonal antibodies in clarifying the diagnosis of tumours of uncertain origin has been established. A retrospective study of the effect on management and prognosis of 82 patients referred for this clarification has been performed The original diagnosis was changed to non-Hodgkins lymphoma (NHL) in 28 cases labelled carcinoma, 1 labelled germ cell tumour and 1 Hodgkins disease; and to melanoma in 3. Three diagnosed as NHL became carcinoma. Mean age of patients was the same in carcinoma and lymphoma groups – 62 years. Mean follow-up for lymphomas was 25.6 months, carcinomas 19 months, others 17.9 months. Sites of disease were mainly in lymph nodes, thyroid, skin, gut and bone. Management was changed in 25/28 patients whose diagnosis became NHL from carcinoma, 2/3 of those changed from NHL to carcinoma and in the 3 changed to melanoma. Surgery was either modified or abandoned in 6 cases and chemotherapy added or modified in 14. Seven patients rediagnosed as high grade NHL received aggressive chemotherapy. The doses of radiotherapy given were reduced in 13/32 and increased in 3/32 patients as a result of change of diagnosis. The response to treatment in the originally and newly diagnosed NHL groups respectively were CR 60% and 54%; PR 20% and 17%; PD 20% and 23%. The responses in the carcinoma and other tumour groups were similar with CR-35%, PR-41%, SD-6%, and PD-18%. However median survival in these patients was only 15 months compared to 24 months for the lymphoma group as a whole (logrank $P<0.05$). This panel of monoclonal antibodies provides a clinically useful investigation upon which to base the treatment and predict the prognosis of patients with tumours of uncertain histological type.

### Conference lecture

### Alpha transforming growth factors in normal and malignant human mammary epithelial cells

D.S. Salomon, S. Bates, R. Dickson, N. Kim, E. Valverius, M. Lippman & W.R. Kidwell

*Laboratory of Tumour Immunology and Biology and Medicine Branch, National Cancer Institute, National Institutes of Health, Bethesda, Maryland, 20892, USA.*

Transforming growth factor alpha (TGFα) has been circumstantially implicated in the autocrine-stimulated growth of a number of rodent and human tumour cells. In addition, the enhanced expression of several oncogenes, such as *ras*, can increase the synthesis and secretion of TGFα whereas increased expression of the *myc* oncogene can hypersensitize cells to the biological effects of TGFα and epidermal growth factor (EGF). TGFα functionally and structurally resembles EGF since it is able to compete with EGF for binding to EGF receptors and since it is capable of stimulating the anchorage-independent growth (AIG) of nontransformed cells, such as NRK cells, in soft agar. Because overexpression of the Harvey (Ha)-*ras* and *myc* oncogenes occurs in a subset of human breast cancers, the role that TGFα might perform in these tumours assumes increased importance. Moreover, TGFα may function as a proximal effector for certain mammotrophic hormones such as oestrogen $E_2$. Conditioned medium (CM) from several human breast cancer cell lines, including MCF-7, T47, ZR-75-1, and MDA-MB-231, contains a high molecular weight (30 kDa) TGFα activity that can compete with EGF in an EGF radioreceptor assay (RRA), which can stimulate the AIG of NRK cells in soft agar and which can react with monospecific TGFα antibodies in a competitive radioimmunoassay (RIA) or following radioimmunoprecipitation. The CM levels of immunoreactive TGFα range from 0.5 to 40 ng $10^{-8}$ cells, woth the MDA-MB-231 cells exhibiting the highest levels and ZR-75-1 cells possessing the lowest levels. Treatment of oestrogen-responsive MCF-7, T47-D, and ZR-75-1 cells with $E_2$ ($10^{-8}$ M) produces a 2- to 5-fold increase in the CM levels of biologically active and immunoreactive TGFα. Following Northern blot analysis with a human TGFα cDNA probe, a specific 4.8-kb TGF mRNA species can be detected in the poly $(A)^+$ RNA isolated from the human breast cancer cell lines at levels proportional to the amounts of secreted TGFα in the CM. Treatment of MCF-7 cells with $E_2$ *in vitro* for 6 h leads to a 2- to 3-fold induction in the level of TGFα mRNA expression. Furthermore, withdrawal of oestrogen from nude mice bearing oestrogen-dependent MCF-7 tumours results in a decrease in TGFα mRNA levels within 2 to 3 days. Polyclonal antibodies against human TGFα can inhibit the AIG of MCF-7 cells in soft agar. Transfection of a v-Ha-*ras* oncogene into MCF-7 cells abrogates the *in vitro* and *in vivo* growth-promoting effects of $E_2$ and augments the production of TGFα by 3- to 4-fold with a loss of $E_2$ induction. TGFα protein and mRNA have also been detected in ~50 to 70% of primary human breast tumours. TGFα is not restricted to malignant human mammary epithelial cells. For example, primary cultures of normal human mammary epithelial cells (HME) or HME cells that have been immortalized with benzo-a-pyrene (A1N4) contain biologically and immunologically active TGFα in their CM and express a 4.8-kb TGFα mRNA. Following Southern blot analysis, no evidence for amplification or gross rearrangements of the TGFα gene was observed in any of the breast cancer cell lines or in the A1N4 cells. These results, in conjunction with the observation that comparable TGFα species have been identified and purified from human milk, suggest that TGFα is functioning as a mitogen for normal proliferating and neoplastic human mammary epithelial cells.

### Gynaecological cancer

### Radiobiology of human squamous cell carcinoma of the cervix

L.R. Kelland, K.S. Tonkin, L. Burgess & G.G. Steel

*Institute of Cancer Research, Sutton, Surrey, UK.*

The clinical management of carcinoma of the cervix consists primarily of surgery and radiotherapy employing both external beam high dose-rate and intracavitary low dose-rate regimes. From human cervix carcinoma biopsies we have established four new continuous cell lines which, in addition, are serially transplantable as xenografts in nude mice. We are using these to investigate the *in vitro* and *in vivo* radiobiological properties of this disease.

*In vitro* studies have been performed using single cell suspensions of tumour cells, plated out and irradiated with $^{60}$Co λ-rays at high dose-rate (150 cGy min$^{-1}$) and continuous low dose-rates (3.2, 1.6 cGy min$^{-1}$). Cells were held at 37°C in a 5% $CO_2$, 5% $O_2$, 90% $N_2$ atmosphere throughout irradiation and assayed for survival by *in vitro* cloning. For *in vivo* studies, tumours were grown as xenografts over the dorsal spine of nude (nu/nu) mice and the tumours were irradiated at dose-rates of 75 and 5 cGy min$^{-1}$ with lead shielding of the body. The end-point used was tumour growth delay.

Results showed that both *in vitro* and *in vivo*, variations in acute survival parameters and wide variations in low dose-rate sparing occurred between the lines. The most radioresistant line HX156 showed substantial dose-sparing *in vitro* (Dose Reduction Factor of 1.51 at the 1% survival level for 150 *versus* 1.6 cGy min$^{-1}$) whereas for HX151 this was only 1.14. The large amount of sparing in HX156 was also reflected *in vivo* with tumour growth delays of 4.0 and 1.1 for 12 Gy total dose administration at dose-rates or 75 or 5 cGy min$^{-1}$ respectively.

### Neo-adjuvant bleomycin, methotrexate and CCNU in advanced radically inoperable squamous cell carcinoma of the vulva

K.R. Durrant, C. Mangioni, M. George, A. La Cave, M.E.L. Vandeburgh, N. Rotmenz & J.B. Vermorken

*EORTC Gynaecological Cancer Cooperative Group, Churchill Hospital, Oxford, UK.*

Radical vulvectomy is the treatment of choice for squamous cell carcinoma of the vulva. Down-staging of inoperable disease by radiotherapy or chemotherapy is impeded by the age and poor general condition of these patients. Therefore low dose chemotherapy with bleomycin, methotrexate and CCNU has been studied in 23 patients with radically inoperable vulval cancer for clinical efficacy, operability after chemotherapy, and side-effects profile. All had histologically verified squamous carcinoma, inoperable, measurable, and not pretreated. Patients with distant metastases or severe coexistent disease were excluded. Chemotherapy was given in a 6 week cycle using a complex schedule of dose modification for toxicity: BLM 5 mg i.m. days 1–5, then days 1 and 4 each week, CCNU 40 mg p.o. days 5–7, MTX 15 mg p.o. days 1 and 4 each week. A minimum of one cycle was given and repeated four times or until response.

Eighteen patients were fully evaluable, 11 with primary tumours, 7 with recurrent disease. The mean age was 75 years, mean performance status was 1.

| *Response* | | *Operability* | |
|---|---|---|---|
| Complete | 2 | Not operable | 11 |
| Partial | 10 | Surgery performed | 4 |
| No change | 4 | Not evaluable | 1 |
| Progression | 2 | Too early to assess | 2 |

Side effects were more severe than expected from pilot studies with frequent stomatitis, nausea and infection.

### Advanced and recurrent cervical cancer – Active chemotherapy

G. Constantine[1], C. Meanwell[1], G. Blackledge[1], J. Mould[1], A. Chetiyawardana[1], D. Spooner[1], T. Latief[1], F. Lawton[1], J. Kavanagh[1], J. Tobias[2], M. Patterson[3], M. Sokal[4] & C. Alcock[5]

[1]*Clinical Trials Unit, Queen Elizabeth Hospital, Birmingham B15 2TH;* [2]*University College Hospital, London;* [3]*Northern General Hospital, Sheffield; and* [4]*General Hospital, Nottingham; and* [5]*Churchill Hospital, Oxford, UK.*

Thirty-five evaluable patients with recurrent or disseminated cervical cancer were entered into a prospective trial of bleomycin (30 mg infused over 24 h), followed by *cis*-platinum (50 mg m$^{-2}$ bolus) and ifosfamide (5 g m$^{-2}$ infused over 24 h), with concomitant hydration (total 10 l over 3 days) and mesna (8 g m$^{-2}$ given during and for 12 h following ifosfamide). Patients received between 1 and 8 courses (mean = 4.3). Twenty-seven of 35 had previously received radiotherapy (RT).

Twenty-five (72%) objective responses were seen. Seven women had a CAT span complete response. Most patients noted a subjective improvement in disease related symptoms. Seven patients went onto further RT to consolidate the response. Response duration to date varies between 6 and 35 weeks with chemotherapy alone, 7 patients having a continuing response. All patients experienced alopecia and nausea/vomiting. Fifteen (43%) needed one or more blood transfusions, 6 (14%) developed a septicaemia, 7 (20%) developed grade 1 or 2 ifosfamide encephalopathy, 2 patients developed renal damage and there was 1 death from septicaemia.

Twelve further patients with advanced disease have been treated in a neo-adjuvant setting prior to RT, 70% having a >50% reduction in tumour size within 2 courses. These data indicate that BIP may be used for effective palliation and debuling in around 70% of patients with advanced or recurrent cervical cancer. A multi-centre randomised trial has been launched to determine whether neoadjuvant BIP improves survival in patients with inoperable cervical cancer.

### Evaluation of toxicity in patients with ovarian carcinoma treated by intensive combined modality therapy

S. Myint, J.A. Green, R.D. Errington, A.J. Slater & H.M. Warenius

*CRC Department of Radiation Oncology, University of Liverpool, UK.*

Forty-six high risk patients with ovarian carcinoma were treated by intensive combined modality therapy. Thirty-three patients had TAH/BSO completed and after surgery 21 patients had bulky residual disease. All 46 patients received combination chemotherapy with *cis*-platinum and cyclophosphamide following surgery and 18 patients had complete abdomino-pelvic radiotherapy. Thirty-eight patients (65%) were in complete remission including 10 patients who had pathological CR. Overall response was 78% and 55% of patients were alive at 30 months.

Toxicity of the intensive combined modality therapy is reviewed. Data on haematological, GI tract, renal and hepatic toxicities are presented. In all 18 patients who had had all 3 treatment modalities, the toxicity was moderate but tolerable. There were no toxic deaths related to treatment.

Intensive combined modality therapy is necessary in high risk patients to improve results, both in terms of local control and survival, but toxicity from such therapy should be borne in mind.

### The assessment of bladder dosage in intracavitary therapy of carcinoma of cervix

R.D. Hunter[1] & H.M. Notley[2]

*Departments of [1]Radiotherapy and [2]Medical Physics, Christie Hospital and Holt Radium Institute, Manchester M20 9BX, UK.*

Pelvic dosimetry in intracavitary therapy has always been a problem because of difficulties in identifying easily the position of individual organs and of expressing dosage to them in view of the inhomogeneity of the exposure. As a compromise ICRU 38 defined bladder dosage by reference to the post wall of a Foley catheter balloon. Previous data from this hospital demonstrated that there was no correlation between this ICRU bladder reference dosage and the maximum dose to the posterior bladder wall in patients undergoing intracavitary therapy. This study defined a vertical plane through the upper vaginal applicator in which the maximum bladder base dosage was likely to occur. Subsequent studies failed to find any correlation between the distance from the mid plane of the vaginal ovoids and the posterior bladder wall and (1) stage, (2) age or (3) size of vaginal applicators. This distance can be calculated by CT scanning but this is impractical in routine clinical practice. A new technique will be described in which the bladder base and posterior wall can be defined in a patient undergoing intracavitary therapy using conventional radiography. A group of 20 patients have been investigated in whom the new technique has been correlated with CAT scanning. The use of the new technique on conventional radiographs allows the bladder to be visualised successfully and helps to explain many of the problems encountered previously.

### Conference lecture

### The strategy for new drug development

M.F.G. Stevens

*Department of Pharmaceutical Sciences, Aston University, Birmingham, UK.*

Genes coding for proteins which confer multiple drug resistance and the ability to repair DNA alkylation lesions provide the cancer cell with the means to subvert intervention by many antitumour drugs. Similarly, amplification of genes coding for the protein structures of vital enzymes (e.g., dihydrofolate reductase or thymidylate synthetase) provide the cancer cell with the ability to develop resistance to drugs targeted to these enzymes, and others. A similar outcome is likely to frustrate the efforts of drug designers developing new generations of agents targeted to oncogene protein products (e.g. growth factors and growth factor receptors).

A new strategy for drug development will focus on the design of agents which can recognise, in a sequence-specific manner, key oncogene nucleotide sequences and either silence the transcription of the gene or inhibit translation of the messenger RNA sequence. The structures of prototype molecules in this new class will be discussed and prospects for success of this strategy reviewed. The Cancer Research Campaign has established a programme with the resources to bring novel drug moieties into clinical trial: these new moieties will be selected on different biological criteria than hithertofore.

### Brain tumours

### Growth failure following irradiation in children with brain tumours

E.A. Livesey, C.G.D. Brook, A.C. Whitton, J.A. Britton, J.S. Tobias & H.J.G. Bloom

*Middlesex, University College and Royal Marsden Hospitals, London, UK.*

We have studied the growth and endocrinology of 120 children from a cohort of 140 who are in clinical remission following treatment of a brain tumour. All received cranial irradiation, 66 spinal irradiation and 32 adjuvant chemotherapy. Mean age at treatment was 6.3 years (0.8–15). Mean follow-up since completing radiotherapy was 8.5 years (1.2–26).

Thirty-two had completed their growth before endocrine assessment. Fourteen who had received spinal irradiation had a mean final standing height standard deviation score (SDS) of 1.82 below the mean for the normal population and mean final sitting height (reflecting spinal growth) SDS −2.94. Eighteen treated with cranial irradiation alone had a final height SDS of −0.94 and final sitting height SDS of −1.21. Hence spinal irradiation has a slightly significant effect on final height.

Low height velocity, reflecting the rate of growth, was observed in 94 children followed prospectively, and growth hormone insufficiency (in response to insulin induced hypoglycaemia) was found in 83 of 85 children assessed. The incidence of growth hormone insufficiency is clearly higher than has previously been reported.

Forty children were treated with growth hormone and all responded with improved height velocity. Delay in instituting treatment in growth hormone deficient children leads to an irreversible loss of final height prognosis. Prospective follow-up and early intervention are essential to achieve a normal final adult height.

### pH in human brain tumours

R.P. Beaney[1], D.J. Brooks[2], D.G. Thomas[2] & I. Silver[3]

[1]*Queen Elizabeth Hospital, Birmingham;* [2]*National Hospital for Neurological Diseases, London; and* [3]*Department of Pathology, University, Bristol, UK.*

Current teaching would lead us to believe that most if not all tumours have a pH lower than that of normal tissue. Studies using new techniques, e.g., magnetic resonance spectroscopy, have challenged this belief. We studied regional cerebral pH using 2 independent techniques. Using positron emission tomography and continuous inhalation of $^{11}CO_2$ we measured regional cerebral pH in 12 patients with intracranial tumours. This technique allowed us to determine the intracellular pH of both normal and neoplastic brain in a virtually non-invasive fashion. Regional pH in 8 out of 12 tumours was higher than that of normal brain. Mean regional tumour pH for the group as a whole (7.03±0.07) did not differ significantly from that of contralateral cortex (7.00±0.05). Five patients had pH electrode measurements during craniotomy, 2 out of 5 patients had a tumour pH higher than normal brain, though mean regional tumour pH was not significantly elevated for the group as a whole. There is now evidence to suggest that not all tumours are at a pH lower than that of normal tissue. Admittedly the newer techniques involve mean measurements for a volume of more than a cubic centimetre. In future rather than assuming all tumours to be acidic it may be important to establish the pH of individual tumours or different tumour types. This would allow specifically tailored treatment to be given that would exploit any difference in pH to the full.

### CT computerised stereotactic multiple biopsies for low density CT lesions presenting with epilepsy

J.N. Wilden[1] & P.J. Kelly[2]

[1]*Institute of Neurological Sciences, Southern General Hospital, Glasgow, UK; and* [2]*Department of Neurosurgery, Mayo Clinic, Rochester, USA.*

Thirty-five patients presenting with epilepsy alone and a non-enhancing low density lesion on the CT scan underwent computer-assisted CT-guided multiple stereotactic biopsies with stereotactic angiographic control. There was no mortality or morbidity in this series and the diagnostic yield was 97%. Thirty-four patients had low grade intra-axial neoplasms. After an estimation of the pathological extent of the tumour, 3 patients underwent a computer-assisted stereotactic laser resection and 28 patients had radiotherapy.

Multiple serial biopsies offer a method of estimating the pathological boundaries of the tumour in three dimensions. Once these boundaries are known a more rational decision can be made regarding the feasibility of the total versus partial surgical resection of low grade intra-axial neoplasms and can be helpful in planning radiotherapy.

### The influence of field size on the radiation tolerance of the spinal cord: Experimental findings leading to a reappraisal of clinical data

A. Morris[1] & A. Dixon-Brown[2]

[1]*Research Institute and* [2]*Department of Radiation Physics, Churchill Hospital, Oxford OX3 7LJ, UK.*

It is current clinical practice to reduce the total therapy dose as the length of spinal cord irradiated is increased. The early clinical findings of Boden (1948) are frequently quoted in support of this approach, even though his conclusions were based on very limited data. While several subsequent authors have made reference to a field size effect, little or no additional data was presented and precise relationship has been established.

In a recent series of experiments in the rat, 4, 8 and 16 mm lengths of cervical cord were irradiated. The dose related incidence of paralysis within <30 weeks (white matter damage) and for intervals >30 weeks (vascular damage) was assessed. For paralysis within 30 weeks a field size effect was seen, the $ED_{50}$ value increased from 21.5±0.3 Gy for a 16 mm field to 50.98±2.28 Gy for a 4 mm field. However, this effect was largely lost for the later damage; $ED_{50}$ values ranged from 20.0±0.5 Gy (16 mm) to 25.58±2.78 Gy (4 mm), moreover at $\leqq ED_{25}$ no significant field size effect was observed.

From an analysis of data from a paper by Reinhold *et al.* (1974) similar total doses were found to be associated with myelopathy in patients in which ≦10 cm and ≧12 cm of the spinal cord was irradiated. This finding appears to contradict the early data of Boden. The experimental findings and the analysis of more recent clinical data suggests the need for a reappraisal of existing clinical guidelines.

### Further observations on the relationship between drug sensitivity *in vitro* and relapse free interval (RFI) in patients with glioma

J.L. Darling, D.G.T. Thomas, E.A. Paul & C. Twelves

*Institute of Neurology and University College Hospital, London, UK.*

We have previously described a relationship between sensitivity *in vitro* to CCNU or procarbazine (PCB) and RFI in patients with malignant glioma. We report here a larger series with more extensive follow-up. One hundred and fifty-seven patients (63 with grade III and 94 with grade IV gliomas, age range 18–76) were treated with PCB (100 mg m$^{-2}$, p.o. days 1–10), CCNU (80 mg m$^{-2}$, p.o., day 1) and vincristine (VCR, 1.5 mg m$^{-2}$, i.v. day 1) following surgery and a course of adjuvant radiotherapy. Chemotherapy was administered in 12 cycles at 6 weekly intervals. Samples were taken at surgery from 56 patients, cultured and the chemosensitivity obtained to each drug using a $^{35}$S-methionine uptake assay. By comparison of the $ID_{50}$ obtained for each drug to a large training set of cultures derived from malignant gliomas it was possible to designate each patient's culture as either sensitive or resistant to each of the drugs used clinically. Those with cultures which were sensitive *in vitro* to PCB and/or CCNU had a more favourable prognosis than those whose cultures were not (Lee-Desu Statistic = 19.2; df = 1; $P < 0.0001$). Such a chemosensitivity test may be useful in selecting chemotherapy for patients with glioma.

### New developments

### Free flap reconstruction for head and neck cancer

M.D. Brough

*University College Hospital, London, UK.*

The first free flap transfer with microvascular anastomoses was performed in 1972. During the last fifteen years many different flaps of composite tissue including skin, muscle, bone and bowel have been described as suitable for transfer. The quality of instruments and techniques involved in this surgery have improved during this time and the success rate of transfer has now reached a high level.

Free tissue transfer is particulary appropriate in the management of some head and neck cancers. It provides a single stage reconstruction following extensive excisional surgery. The quality of the result is often better than that hitherto achieved and the distant donor site has a low morbidity.

### Brachytherapy for carcinoma of the oesophagus

K.M. Pagliero & C.G. Rowland

*Royal Devon & Exeter Hospital (Wonford), Exeter, UK.*

Disappointment with external beam irradiation for palliation of malignant dysphagia has led us to look at brachytherapy as an alternative. We have designed an applicator which can be sited endoscopically under radiological control to treat oesophageal lesions using the Selectron (Nucletron, Holland) to load 48 Caesium 137 sources as a 12 cm line source. In appropriate cases we can treat the entire oesophagus in two immediately consecutive applications. In our pilot study 72 patients deemed inoperable on grounds of unfitness or unresectability underwent brachytherapy. Two patients could not tolerate the treatment. Four-fifths of those treated had useful improvement in swallowing. Subsequent recurrences in previous responders were retreated. Twenty per cent of patients ultimately required intubation. The side effects were few: mild oesophagitis occurred in a few; 1 patient developed a radiation stricture which responded to bougienage; 1 patient with tracheal involvement developed a fistula and would not now be so treated. Adenocarcinoma responded almost as well as squamous cell carcinoma. Comparison with our reported experience with intraluminal stenting shows that brachytherapy has resulted in the elimination of mortality, considerable reduction in hospital stay and complications and an increased survival.

### Endoscopic laser palliation for advanced gastrointestinal cancer

S.G. Bown, L.A. Loizou, K. Matthewson, H. Barr, P.B. Boulos & C.G. Clark

*National Medical Laser Centre, Department of Surgery, University College Hospital, London, UK.*

Palliative treatment of malignant dysphagia aims to optimize swallowing as quickly as possible with the minimum of general distress to these seriously ill patients. We treated 34 patients endoscopically with the NdYAG laser who were considered unsuitable for surgery due to advanced malignancy or other major pathology or in whom previous surgery had been unsuccessful. Two also had radiotherapy. Significant improvement was achieved in 29 (85%). On a scale of 0–4 (0 = normal swallowing; 4 = dysphagia for all fluids), the mean improvement was 1.7, with 25 patients (74%) able to swallow most or all solids within a few days of completion of a course of treatment. Failures were due to inappropriate patient selection (3) or laser related perforation (2). The mean survival for the whole group was 19 weeks (range 2–44). Early recurrence (mean 5 weeks) in 13 patients due to exophytic tumour responded to repeat laser therapy, but late recurrence (mean 10 weeks) in five patients due to extensive tumour or laser related fibrous stricturing required insertion of a prosthetic tube. Endoscopic laser therapy gives rapid relief of dysphagia, but recurrence is common. A combination of laser therapy with radiotherapy or chemotherapy could give the best long term results in these patients.

Endoscopic laser therapy was also used for relief of bleeding, obstruction, diarrhoea and incontinence in 17 patients with advanced rectal cancers. Significant improvement was achieved in 15 (88%) with minimal morbidity and no complications. Of 14 who have died (after a mean of 15 weeks) only three had recurrent symptoms requiring further intervention.

### Photochemotherapy: Tumour response and early skin reaction

D. Gilson, D. Ash, I. Driver, J. Feather & S. Brown

*Departments of Radiotherapy, Medical Physics and Biochemistry, Leeds University, UK.*

Ten patients with cutaneous or sub-cutaneous recurrences of malignant disease have been treated with photochemotherapy in Leeds. Many have had multiple lesions and have been treated with varying doses of photofrin II and light. Thirty-three lesions have been treated with 1.0, 1.5 or 2.0 mg kg$^{-1}$ of photofrin II followed 48–72 h later by surface illumination using red light (630 nm) produced by an argon/dye laser (dose rate 40–172 mW cm$^{-2}$). A range of doses from 25–100 J cm$^{-2}$ was delivered.

Only complete responses of the tumour were scored together with severe skin reaction which was associated with the formation of a black eschar.

With increasing doses of photofrin II and increasing doses of light irradiation complete tumour response steadily increased. This was, however, almost paralleled by the incidence of skin necrosis suggesting that there is a relatively low therapeutic ratio for superficial skin illumination in photochemotherapy. Skin necrosis caused little or no discomfort to patients, however, and eventually healed in all cases.

The use of interstitial light delivery by implantation of optical fibres has been initiated to try and improve the therapeutic ratio and this may also increase the range of lesions amenable to treatment.

### Induction of necrosis in an experimental mouse lymphoma using modifiers of tumour oxygenation and a bioreductive drug.

C.H. Du Boulay, G.E. Adams, I.J. Stratford, S. Butler & J. Nolan

*MRC Radiobiology Unit, Chilton, Didcot, UK.*

Oxygenation in even small tumours can be substantially less than in surrounding normal tissues. This can be exploited in the design and application of drugs which rely for their anti-tumour effect on reductive activation. The efficiency of such agents can be greatly increased by substances that further decrease tumour oxygenation.

This paper describes a histological study of the induction of necrosis and shrinkage of small infiltrated lymph nodes in an experimental T-cell mouse lymphoma. Tumour-bearing mice were treated with the vaso-active drug, hydralazine, either alone or in combination with nitroheterocyclic bioreductive drugs including the bifunctional compound RSU 1069. Normally, histological sections of infiltrated lymph nodes show no necrosis although studies with tritium-labelled misonidazole show that some degree of hypoxia is present. Treatment with either single or multiple doses of hydralazine caused some tumour necrosis. Combinated treatment with both hydralazine and RSU 1069 caused massive necrosis and tumour shrinkage. In some cases, no residual viable tumour cells were visible in the nodes. Extensive reduction of tumour load also occurred in infiltrated liver and spleen.

### Vascular collapse: A component of tumour therapy?

J.C. Murray, V.S. Randhawa & J. Denekamp

*CRC Gray Lab., Northwood, UK.*

Tumours are frequently poorly nourished due to inadequate vascular supply. This deficiency may have important consequences both for chemotherapy, where delivery of drugs to distant cells may be suboptimal, and for radiotherapy, as hypoxic cells are known to be radioresistant. We are interested in whether this deficiency can be exploited to therapeutic advantage, and are currently examining the effects of various forms of therapy on vascular structure and function in experimental tumours.

Vascular function has been assessed in the SaFA murine sarcoma using a fluorescent dye perfusion technique. Hoechst 33342 was injected i.v. into tumour-bearing mice and allowed to circulate for 1 min. After sacrifice, tumours were removed, frozen and sectioned at 6 $\mu$m. The distribution of Hoechst dye was assessed by Chalkley point counting of sections viewed microscopically under epi-fluorescence. Sections were also stained using the immunofluorescent technique with an antibody to mouse laminin to delineate the basement membrane of tumour blood vessels, allowing the estimation of total vascular volume. Using these techniques we examined the effects of chemotherapy, in rhe form of combined melphalan and misonidazole (MISO), as well as varying single doses of X-rays (10–40 Gy), and attempted to correlate vascular effects with tumour response in terms of regrowth delay.

A combination of melphalan and MISO which induced a 2 week growth delay also caused a profound decrease in effective vascular volume within 24 h after treatment. This vascular effect was shown to be largely due to the MISO, although MISO alone did not affect tumour growth. Doses of X-rays which induced a growth delay similar to that from the combined drugs did not decrease vascular volume significantly at any time after treatment, as assessed by Hoechst perfusion alone.

We conclude that the contribution of vascular collapse to tumour growth delay may vary with different forms of therapy.

### Rectal tumours

### External beam radiotherapy for rectal adenocarcinoma

R.E. Taylor[1], G.R. Kerr[1] & S.J. Arnott[2]

[1]*Department of Clinical Oncology, Western General Hospital, Edinburgh; and* [2]*St. Barthelomew's Hospital, London EC1, UK.*

Between January 1974 and December 1983, 243 patients with rectal adenocarcinoma were treated with external beam pelvic radiotherapy; 74 were treated with radical radiotherapy for inoperable or recurrent disease; 145 with advanced pelvic tumours or metastases were treated palliatively; 24 with small volume residual pelvic tumour or who were felt to be at high risk of pelvic recurrence following radical resection received postoperative radiotherapy. Between 1974 and 1977 46 patients (radical: 16, palliative: 30) received 5 fluorouracil (5 FU) 250 mg daily with each fraction of radiation. Actuarial survival at 2 years was 29.0% for inoperable, 31.6% for recurrent, 10.2% for palliative and 62.1% for postoperative patients. Survival was significantly better for patients with small ($\leq$5 cm) tumours ($P<0.001$).

Complete tumour regression was observed in 38% of radically treated and 24% of palliatively treated patients, and partial regression in 56% of radically treated and 58% of palliatively treated patients. Survival was significantly better for patients responding completely to radiotherapy ($P<0.001$). Long term local control was more commonly observed for small tumours. Symptomatic response was observed in 75% of radically treated and 77% of palliatively treated patients.

The addition of 5 FU did not appear to improve survival or local control. Fifty-eight per cent of patients treated postoperatively remained free of local recurrence.

### Benefits expected from conformation radiotherapy in the treatment of pelvic tumours

D. Tait[1], A. Nahum[2], C. Southall[1] & J.R. Yarnold[1]

*Departments of* [1]*Radiotherapy and* [2]*Physics, Royal Marsden Hospital, Surrey, UK.*

In cancers of the bladder, prostate and cervix, the radiation dose that can be delivered by conventional beam arrangement is limited by bowel tolerance. The scope for adapting the shape of the high dose volume to conform more closely to that of the tumour volume is being investigated in (15) patients with bladder cancer by exploiting the independent collimator movement available on the Philips SL-25 linear accelerator. One cm CT slices, taken through the pelvis with the patient in the treatment position, are used to reconstruct tumour, rectal and bowel volumes included in the treatment length. From the CT scans, a conventional small volume treatment plan is devised using a three field arrangement. The same treatment length is then divided into three horizontal segments, the dimensions of which are adjusted to encompass tumour volume with maximum exclusion of normal bowel. Keeping the same gantry angles, three fixed fields are used to cover the segmented treatment volume by employing the collimator sweep in the long (Y) axis, combined with the cross axis collimator facility. This is a simple manoeuvre which could be implemented immediately on the Philips SL-25. Cumulative volume dose distributions of a typical conformal plan compared to the corresponding conventional distribution show a 25% reduction in the overall treatment volume (defined by the 50% isodose) which includes up to 50% reduction in the volume of rectum and bowel in the high dose zone. The potential therapeutic gain of the conformal plan will be discussed.

### Louise Buchanan Memorial Lecture

### The curative treatment of rectal cancer with radiotherapy (RT)

J.-C. Horiot

*Cancer Institute Georges-François Leclerc, 21034 Dijon, France.*

Few rectal carcinomas can be satisfactorily treated with surgery alone, *viz.* medically operable patients with Dukes/Gunderson Sosin A, $B_1$ treated by anterior resection. All other clinical presentations will benefit from RT as part of, or as the single treatment. This provocative statement is now supported not only by a number of historical series, but also by at least three randomized trials in the United States and Europe (GITSG 71-75, EORTC 40761, NCCTG-Mayo).

Loco-regional failure rate is significantly reduced by either pre-operative or post-operative RT. Trends and even improvement in survival are obtained in subsets of patients of the RT arms.

Limited, accessible, exophytic rectal adenocarcinomas are indications for intracavitary RT techniques (X-ray 50 kV contact RT with or without interstitial Ir 192 brachytherapy): a 90% cure rate is achieved in J. Papillons' work as well as in our own material in these cases, with sphincter preservation. We have described a clinical staging for non-fixed rectal cancers: a significant correlation ($P=0.001$) is obtained between the staging criteria and disease free survival rate.

The latest development in this area is the concept of conversion of a classical indication of abdomino-perineal surgery into a sphincter saving management: 2 months after a 30 Gy in 10 fractions and 2 weeks external radiotherapy is given, a decision is taken either to proceed with abdomino-perineal surgery, or with low anterior resection, or with a boost dose of RT (usually with interstitial Ir 192):

Both J. Papillon's experience and our own data demonstrate that 60% of the patients with moderately advanced low rectal cancer can be cured with the preservation of a functional sphincter.

### Posters

### Epirubicin *versus* mitozantrone in advanced breast cancer

S.N. Larsson, G. Blackledge, A. Chetiyawardana, T. Latief, J. Mould & M. O'Brien

*CRC Clinical Trials Unit, Birmingham, UK.*

A trial was designed to investigate two new anthracycline cytotoxics, alone and in combination, in advanced breast cancer.

Published data suggest that factors predicting poor response and survival exist in patients starting chemotherapy for advanced disease. In our own multivariate analysis, interval of under two years from diagnosis ($P=0.006$) and presence of visceral metastases ($P=0.029$) were significant. It may be appropriate to reserve aggressive combination chemotherapy for patients exhibiting poor prognostic factors.

This trial separates patients with objectively assessable disease into groups: a 'high-risk' group below age 70 years with visceral metastases or an interval of under two years between diagnosis and starting chemotherapy, and a 'low-risk' group of patients falling outside these criteria. To be accepted, patients must be off hormone therapy and must not have had previous chemotherapy. The 'high-risk' group are randomised between epirubicin, mitozantrone and adriamycin, each in combination with methotrexate and cyclophosphamide as an i.v. pulse every 3 weeks. The 'low-risk' group are randomised between epirubicin, mitozantrone and adriamycin as single agents every 3 weeks. Weighted randomisation is used so that 40% of patients receive the novel agents and 20% receive adriamycin. Objective assessment of response rates, duration and toxicities are made using WHO criteria. By March 1st, 1987, 90 patients were randomised: 70 into the 'high-risk' group and 20 into the 'low-risk' group. It is hoped to close the trial when 250 patients have been randomised. The rate of accrual has been fairly constant 10 patients per month.

### Sequential chemotherapy, surgery and radiotherapy in locally advanced breast cancer

I.R. Campbell, J.A. Green, R.D. Errington, S.J. Leinster, S. Myint & H.M. Warenius

*CRC Department of Radiation Oncology, Clatterbridge, Wirral, UK.*

Thirty-seven patients with T3b, T4, N2 or N3 advanced local breast cancer were treated with 3–6 cycles of vincristine $1.4\,mg\,m^{-2}$, doxorubicin $40\,mg\,m^{-2}$ and cyclophosphamide $600\,mg\,m^{-2}$ i.v. with an overall response rate of 67%. Fourteen patients had received a trial of hormone therapy, but no patient had been given prior cytotoxic chemotherapy or radiation therapy. In no case was chemotherapy discontinued on account of toxicity. Where gross disease remained (>3 cm), mastectomy (10 patients) or local excisions (5 patients), was carried out, and 32 patients then received radiotherapy, 57 Gy in 24 fractions to the breast with boost to the tumour site. Complete clinical remission was achieved in 19% of patients after chemotherapy, in a further 30% after surgery, and in a further 27% by radiation therapy, giving an overall remission rate of 86% and a total complete remission rate of 76%. Of the 10 patients with N3 disease, half were dead within 1 year. The survival rate in the entire group was 50% at 2 years. The median time to relapse in the 28 complete responders was 17 months. Local palliation, defined as absence of pain, ulceration or an enlarging mass was achieved in 92% of the total number of patients, and in 60% of the patients surviving 2 or more years from the start of treatment. This an effective and tolerable approach for a sub-group of breast cancer patients with complex management problems.

### Medroxy progesterone acetate: Variation in serum concentration achieved with three comercially available preparations.

A.D. Stockdale & A.Y. Rostom

*St. Luke's Hospital, Guildford, UK.*

Twenty-nine females with metastatic or locally recurrent carcinoma of the breast were treated with 1 g medroxyprogesterone acetate (MPA) daily by mouth. This was used as a second or third line treatment. Serum concentration of MPA was measured over a 28 day period. We have demonstrated a significantly greater area under the concentration time curve, peak and steady state MPA concentration, for provera 100 mg and 200 mg (Upjohn) than farlutal 500 mg tablets (Farmitalia). Relative bioavailability of preparations should be considered when prescribing or assessing treatment results when MPA is used.

### Cyclical sequential hormonochemotherapy for the treatment of advanced breast cancer

M.W. Ghilchik, M.J. Reed, N. Shaikh & P.A. Beranek

*Breast Clinic, St. Mary's Hospital, Praedt St., London W2, UK.*

Endocrine or chemotherapy is widely used in the treatment of advanced breast cancer. In order to improve the relatively constant response rates to such therapy, however, it has been proposed that endocrine and chemotherapy should be combined and we are currently investigating the use of cyclical sequential hormonochemotherapy (CySHoC). Hormone therapy was initiated with ethynyloestradiol ($10\,\mu g\,day^{-1}$) for 1 week after which patients received medroxyprogesterone acetate ($500\,mg\,day^{-1}$) for 2 weeks. At the end of the first 3 week period patients received a bolus injection of vincristine (2 mg) and an infusion of adriamycin (50 mg). The hormone therapy was then repeated after which an infusion of cyclophosphamide (500 mg), methotrexate (50 mg) and 5-fluorouracil (500 mg) was given. This treatment formed 1 double cycle and patients received 3 double cycles of CySHoC in all. So far 34 out of 40 patients with advanced breast cancer entered into this trial, irrespective of ER status, and completed 3 double cycles of CySHoC (85%). Of these 34 women, 16 (47%) showed a complete response, according to UICC criteria, 15 (44%) a partial response with only 3 (9%) failing to show any response. The disease free interval for patients showing a complete response (22+7 months) was significantly longer ($P<0.001$) than for those showing only a partial response (5+4 months).

It is concluded that the use of cyclical sequential hormonochemotherapy may offer significant advantage for the treatment of advanced breast cancer than the use of endocrine or chemotherapy alone.

### A simple, effective approach to the infusion of epirubicin

S.R. Ebbs, J.A. Saunders, J.V. Roberts, T. Bates & M. Baum

*Departments of Surgery, Kings College Hospital, London and William Harvey Hospital, Ashford, Kent, UK.*

Anthracycline acute and chronic toxicity depends upon the peak plasma dose of the drug, whilst response rate appears to be independent and may be improved by prolonged exposure to low doses.

Infusion therapy has previously been considered difficult. However, by inserting Hickman catheters and maintaining them without routine dressing and only flushing them weekly with normal saline, we have reduced both cost and time. Rather than heavy, expensive mechanical pumps, infusions through the catheters have incorporated 'Travenol Infusors'. These are small, light disposable cylinders. Both the force to propel and a reservoir for the solution are provided by an elastomeric balloon.

To determine the merits of this method we are conducting a randomised trial of weekly epirubicin, given either by 24 h infusion or bolus injection. So far 33 patients with advanced breast cancer are assessable, 14 have received infusion therapy using a total of 149 infusors. There have been no mechanical failures by the infusor.

| *Toxicity* | *Infusor (n=14)* | *Bolus (n=19)* |
|---|---|---|
| Myelosuppression | 1 | 3 |
| Nausea and vomiting | 2 | 9 $P<0.01$ Fishers exact test |

Infusors reduce acute toxicity; treatment time has decreased and needle phobia and the risk of extravasation have not been encountered. There have been no cases of cardiotoxicity. Infusion anthracyclines offer safe, less toxic chemotherapy with an improved quality of life.

### A high dose tamoxifen regimen

N.A. Shaikh & M.W. Ghilchik

*Breast Clinic, St. Mary's Hospital, Praed St., London W2, UK.*

Twenty-five postmenopausal patients (age range 63–90 years, mean 73 years) with locally advanced breast cancer (stage III and IV) were treated with a high dose of tamoxifen of 100 mg daily for a week, followed by a maintenance dose of 40 mg per day. Compliance was 100%. Tumour tissue from 14 of the 25 patients (56%) was assayed for hormone receptors. The diagnosis was confirmed on a Trucut biopsy. Each patient had a full blood count, biochemical profile, a chest X-ray, ultrasound scan of liver and a bone scan.

At two weeks follow up 8 patients (32%) showed a good response (~50% decrease in tumour size), 9 patients (36%) showed a moderate reponse (~25% decrease in tumour size), but 7 patients (28%) showed a poor or no response. The patients were followed up for a period of 4 months to two years (mean 9.6 months). Seven patients who had not responded at two weeks, did not show any response at 2 years follow-up and in 3 of these 7 non-responders the tumour size increased.

The high dose regimen may help to differentiate early the group of patients who are unlikely to respond to tamoxifen so that they can be treated by an alternate method early rather than late.

### Breast cancer and melanoma – A clinical proposal

J.B. Healy

*St. Luke's Hospital, Dublin 6, Ireland.*

Breast cancer is compared to malignant melanoma of the lower limb. Recurrence patterns for melanoma are either local, within a short distance of the primary growth, or diffuse in the thigh. The latter only occurs when lymphatic glands are invaded and when drainage is obstructed by surgery or tumour mass. A second primary is a separate matter. The same pattern occurs in breast cancer. Chest wall recurrences are due to blocked drainage or dermal back flow and this occurs only when lymph nodes have been invaded. Local spread from surgical excision is as for melanoma. After mastectomy, radiation is only useful if the nodes were invaded. After tylectomy, if the glands were positive, then we need to treat the local lymphatic system but if negative, only the area around the excision.

An attempt is made to justify this view from the literature, from clinical cases and from lymphograms and lymphoscintograms.

**A phase II evaluation of 4′-epi-doxorubicin (Epirubicin) in small cell lung cancer**

J.R. Johnson & D.A.L. Morgan

*Hogarth Centre of Radiotherapy and Oncology, Nottingham, UK.*

Twenty-four patients with small cell lung cancer (SCLC), none of whom had received prior anthracycline treatment, were given Epirubicin as a single agent. In 14, an initial dose of $50\,mg\,m^{-2}$ was used ('low dose'); the others received $100\,mg\,m^{-2}$ ('high dose'). Cycles were repeated at three-weekly intervals with a dose escalation of $25\,mg\,m^{-2}$ for the second or subsequent cycles when deemed clinically appropriate.

Response was assessed by WHO criteria and is tabulated below:

| *Initial dose* | *Progressive disease* | *No change* | *Responders* |
|---|---|---|---|
| $50\,mg\,m^{-2}$ | 11 | 2 | 1 |
| $100\,mg\,m^{-2}$ | 3 | 2 | 5 |

Grade 3–4 toxicity was seen in 12 patients (6 from each group) being due mainly to alopecia (all 12 patients) and thrombophlebitis (6 patients). Gastrointestinal toxicity of this grade was only seen in the 'high dose' group: persistent nausea and vomiting in 2 patients and severe mucositis in 1 patient. On no occasion did treatment have to be delayed or adjusted because of myelosuppression.

The degree of toxicity is acceptable, particularly at the 'low dose'; however the overall response rates indicate that epirubicin is an effective agent against SCLC only at doses at or above $100\,mg\,m^{-2}$.

**Lung carcinoma: Observations on changing phenotype and common biological properties**

G.M. Duchesne, J.J. Eady, J.M. Peacock, M.F. Pera & G.G. Steel

*Institute of Cancer Research, Sutton, Surrey, UK.*

Recent studies of the biological properties of human lung cancer cell lines have suggested major distinctions between the neuro-endocrine small-cell carcinomas (SCC) and other tumour types (non-SCC). This study set out to investigate these properties further and examine the relationships between the cell types.

A total of 13 tumour lines were derived from patients and their morphology, biochemical profiles, intermediate filament (IF) expression, hormone production and ultrastructural features examined. All cell types were found to express endocrine peptides although this was most marked in SCC. Study of the IF showed that all cell types could express both epithelial and neural IF. In addition 3 cell lines have shown alterations in morphology and biochemical properties during successive passages to different cell types. These findings are in keeping with the concept of a common cell of origin, and may have important implications for clinical response to therapy.

**High dose rate brachytherapy in the treatment of lung tumours**

C.G. Rowland & K.M. Pagliero

*Postgraduate Medical School & Royal Devon and Exeter Hospital, Exeter, UK.*

Our initial experience in the treatment of oesophagus carcinoma (120 cases) has proved encouraging in terms of palliation and perhaps surprisingly a number of 1–2 year survivors exist having only had intra-cavitary irradiation. We have used all 48 sources of a low dose rate Selectron to give 15–20 Gy in 1–2 h. Here, we appear to be out of the range of dose rate effect which appears negligible. The catheter diameter, however, limits its use at other sites. In conjunction with Nucletron (UK & Holland) a high dose rate micro-Selectron has been developed passing a 10 Ci iridium source through up to 18 catheters 2 mm in diameter. Our early experience in treatment at various sites particularly lung will be described and the great potential for treatment including cost effectiveness discussed.

**The effect of hemi-body irradiation on immunoglobulin and $\beta_2$-microglobulin levels in drug resistant myeloma**

B.J. Smith, A. Norden, J.S. Tobias, J. Richards & C.R.J. Singer

*University College Hospital, London, UK.*

Hemi-body irradiation (HBI) is an effective treatment for symptoms relating to advanced drug-resistant myeloma and macroglobulinaemia. We have already noted that immunoglobulin levels may fall after treatment with HBI (Tobias *et al*., *Radiother. Oncol.* **3**, 11, 1985) and we have now reviewed 20 patients, all with drug resistant disease, in whom we have complete sequential data analysing pre- and post-treatment immunoglobulin levels. In addition, 7 of these patients have had serial estimation of $\beta_2$-microglobulin ($\beta_2$m) before and after HBI. Fifteen patients had IgG myeloma, 3 IgA and 2 IgM. Fourteen were treated with upper-HBI (U-HBI) (mean dose 7.7 Gy) and 14 had lower HBI (L-HBI) (mean dose 9.3 Gy). Eight patients were treated to both halves of the body. All of these 20 patients had a fall in circulating immunoglobulin level, with an average fall of $13\,g\,l^{-1}$ in the abnormal paraprotein. This effect was similar for both U-HBI and L-HBI fractions. As a percentage of the total abnormal paraprotein measured just prior to HBI treatment, this represents a mean fall of 42% for patients with IgA myeloma, 36% for IgM and 23% for IgG.

Eight patients died of progressive myeloma within an average of 6.7 mth, but without the immunoglobulin returning to pre-treatment levels. Twelve patients lived long enough for their immunoglobulin to return to pre-HBI level; this occurred after a mean of 12.7 mth. By contrast, in 7 patients (all with IgG myeloma) in whom we measured the $\beta_2$m before and after HBI, we could not demonstrate any change in the level, despite the mean fall of $11\,g\,l^{-1}$ in the IgG per HBI fraction. Five patients remain alive and well at periods >2 years from HBI.

**Autologous bone marrow transplantation (ABMT) in acute leukaemia**

J.G. Gribben, A.H. Goldstone, D.C. Linch, J.D.M. Richards & L. Dones

*Joint Department of Haematology, University College and Middlesex Hospitals, London, UK.*

We have applied the same chemotherapy treatment protocol at a single centre to 64 patients with acute leukaemia using combination chemotherapy for bone marrow ablation. The response to high dose chemotherapy and ABMT and its associated morbidity and mortality have been compared in 39 patients with AML and 25 patients with ALL.

Thirty-one patients with AML were treated with ABMT during first complete remission (CR) and 22/31 (70%) remain in unmaintained remission at median follow-up of 29

months. Fourteen of 31 patients have received double grafts and all remain in unmaintained remission, median follow-up 32 months.

Twelve patients with ALL were treated in first CR. Only 3/12 (25%) remain in unmaintained remission at 15, 16 and 36 months post ABMT. Two patients in this group relapsed late after transplant at 32 and 33 months respectively.

In patients treated after first remission of disease only 2/21 (9.5%) remain disease free at 32 and 56 months post ABMT, both patients having ALL in second CR. Seventeen of 21 (81%) have died of recurrent leukaemia.

We find this protocol worthy of further study in AML in first CR, but have abandoned this approach in other groups with acute leukaemia.

**Immunocytochemistry – Its value in cancer diagnosis and management**

E. Heyderman[1] & T. Joannides[2]

*Departments of [1]Histopathology and [2]Radiotherapy, UMDS, St. Thomas Hospital, London, UK.*

The use of immunocytochemical techniques in tumour pathology is well established, and may be essential for precise classification. With the advent of more effective treatment modalities, accurate diagnosis has become even more important for cure, for long term remission, and for effective palliation. Our interest has been predominantly in those tumours which presented with metastatic deposits and an unknown primary site. We have also studied tumours of adjacent organs such as the prostate and bladder, and the prostate and rectum, where it has been difficult on purely morphological grounds to determine the organ of origin. We are in the process of following up 140 patients whose tumours have given rise to problems in histopathological diagnosis. An immunoperoxidase technique has been used for the localisation in formalin-fixed paraffin-embedded sections of a variety of mainly epithelial markers, including carcinoembryonic antigen (CEA), epithelial membrane antigen (EMA), cytokeratin (CAM 5.2), DD9-E7, S100, and prostatic acid phosphatase. Twenty-five were deposits in bone, 20 in lymph nodes, 26 were pleural or pulmonary lesions, 11 were hepatic deposits, 31 were ?prostate ?bladder, and there were 38 from other sites. Most success was achieved in the diagnosis or exclusion of the prostate as the primary site. It was also possible to exclude certain sites such as stomach, pancreas, colon, or kidney, so avoiding unneccesary radiological and/or invasive investigations, with important implications in terms of patient management and care.

**Salvage therapy in relapsed Hodgkin's disease by high dose chemotherapy and autologous bone marrow transplantation (ABMT) – Outcome and indications**

J.G. Gribben, A.H. Goldstone, D.C. Linch, R.L. Souhami, G. Vaughan-Hudson & J.S. Tobias

*Departments of Haematology and Radiotherapy, University College Hospital, UK.*

Thirty-eight patients with advanced relapsed Hodgkin's disease have been treated by high dose chemotherapy and ABMT in our centre. There were 30 males and 8 females. The median age was 29 years. No patient had previous bone marrow involvement. Three patients had failed to achieve complete remission (CR) with first-line alternating chemotherapy (LOPP/EVAP). The remaining 35 patients had received at least two regimens of salvage therapy including localised radiotherapy in 18 patients.

Four patients died of sepsis during the neutropenic phase. Eighteen patients entered CR post ABMT, 4 further patients showed partial response and only two patients have shown no response to high dose therapy. Those who achieve CR have a significant improvement in overall survival over those who do not.

Analysis of BNLI data identifies 3 groups of patients who may benefit from ABMT. Poor prognosis patients who fail first line therapy, failure to alternate firstline therapy and failure of any two sequential modalities of therapy.

Most of our patients were grafted with disease too advanced to achieve a respectable result from ABMT. In the future it seems more appropriate to investigate the role of ABMT *versus* conventional therapy in poor risk patients.

**Optimization of treatment for primary non-Hodgkin's lymphoma of the brain**

A.R. Gershuny, H.J.G. Bloom & A. Horwich

*Royal Marsden Hospital, London and Surrey, UK.*

Primary non-Hodgkin's lymphoma of the brain is a rare disease accounting for less than 2% of intracranial tumours. It appears to behave in a far more aggressive manner than its extracerebral counterpart. Recently there have been reports of a marked increase in its incidence in immunosuppressed individuals; particularly those with the Acquired Immune Deficiency Syndrome.

The natural history and treatment of 15 cases of primary non-Hodgkin's lymphoma of the brain presenting between 1974 and 1986 is reviewed. Most cases showed a rapid response to treatment. Mean survival was only 17 months ranging from 3–55 months with 8/15 patients dying from CNS relapse and 3/15 from incontrolled systemic disease.

The role of surgery, chemotherapy and radiotherapy is discussed. The optimal treatment of this disease remains elusive and the investigation of alternative methods of treatment is warranted.

**A retrospective evaluation of radiotherapy as a curative agent in localised Hodgkin's disease**

B.V. Hudson, G.V. Hudson, M.H. Bennet, K.A. MacLennan & A.M. Jelliffe

*British National Lymphoma Investigation, London, UK.*

An analysis was made of over 750 patients with Hodgkin's disease entered into BNLI studies over the last 15 years. These patients were pathologically or clinically staged I or II with upper half disease, with or without systemic 'B' symptoms, and were histologically graded as being LP, MC NS grade I, and NS grade II.

The disease free survival, survival from 'salvage' therapy, and the overall survival of these patients was determined, together with their relationship to various prognostic factors.

The success of both initial treatment and 'salvage' therapy was found to be related to prognostic factors, with LP and MC histology being related mainly to the presentation lymphocyte count, and nodular sclerosis to the presentation ESR, the histological grade, and the presence or absence of mediastinal involvement.

The overall survival of patients who were over 60 years of age at presentation was significantly worse than that of younger patients. Patients requiring 2nd line treatment who failed to obtain complete remission from it had an extremely poor survival.

**The results of treatment of adenocarcinoma of cervix by radiotherapy**

R.P. Symonds, S.E. Davidson, D. Lamont & E.R. Watson

*Western Infirmary, Glasgow, UK.*

Adenocarcinoma of the cervix has been thought to be less responsive to radiotherapy than the more common squamous tumours. Between 1964 and 1980 1,505 (90.3%) patients with squamous tumours and 95 (5.7%) with adenocarcinomas were treated by radical radiotherapy. The treatment given to either histological type assigned to the same tumour stage was identical.

The actuarial 5-year survival (all stages) of patients with squamous tumours was 53.2% and with adenocarcinomas was 48.5%. When survival is analysed by tumour stage and patient age, survival is very similar for patients with either tumour type.

*Percentage 5-year survival of adeno and squamous carcinoma of cervix*

| | *Adeno Ca* | *Squamous Ca* |
|---|---|---|
| Stage I | 81.8 | 79.6 |
| Stage II | 54.9 | 59.8 |
| Stage III | 29.7 | 38.4 |
| Stage IV | 20.9 | 8.9 |

**Chemotherapy prior to radical radiotherapy for Stage III and IV carcinoma of cervix**

R.P. Symonds, E.R. Watson, T. Habeshaw & S.B. Kay

*Western Infirmary, UK.*

The 5-year survival of patients with Stage III or IVa is 30% and 8% respectively. In order to increase local control and subsequent survival of patients with advanced carcinoma of cervix, 28 patients with Stage III and 15 patients with Stage IVa squamous tumours received 2 pulses of chemotherapy before full dose radical radiotherapy (RT). before chemotherapy, tumour was estimated by ultrasound and during an EUA. *Cis*-platin 50 mg m$^{-2}$, bleomycin 30 mg and vincristine 2 mg i.v. were given on day 1 and day 14. Response to chemotherapy was assessed on day 28 at the start of RT by ultrasound and clinical examination. 42.5 Gy was given in 20 fractions over 4 weeks using 4 meV X-rays to the true pelvis (average field size 15 x 15 x 12 cm) followed by a single Cs 137 insertion (A point dose 33.5 Gy). Twenty-two of 41 (54%) had a partial response to chemotherapy. The 3-year actuarial survival is 52%. Responders to chemotherapy have a better prognosis than non-responders: at 36 months 71% are tumour free compared to 31% of non-responders to chemotherapy ($P=0.016$). Acute and late effects of radiotherapy were not increased.

**Randomised trial comparing abdomino-pelvic radiotherapy with *cis*-platinum in patients with ovarian cancer with no macroscopic disesase after primary surgery**

C.W.E. Redman, F.D. Lawton, D. Luesley, C. Hilton, J. Mould, D. Spooner, T. Latief, K.K. Chan & G. Blackledge

*C.R.C. Clinical Trials Unit, Queen Elizabeth Hospital, Birmingham, UK.*

The objective of this study was to compare radiotherapy, as described by Dembo *et al.* (*J. Rad. Oncol. Biol. Phys.*, **5**, 1933, 1978) with single agent *cis*-platinum in the management of patients with no macroscopic disease after primary surgery. Between November 1981 and November 1986, 37 patients were randomised to receive either pelvic plus abdomino-pelvic irradiation, using the moving strip technique (2250 cGy midplane; total dose to pelvis 4500 cGy) or 5 courses of single agent *cis*-platinum (100 mg m$^{-2}$ i.v. every 21 days). Eighteen patients were randomised into the radiotherapy arm and 19 received chemotherapy. The two groups were comparable for age, tumour histology and differentiation, and FIGO staging (Ic to III). Three of 19 patients, all in the chemotherapy arm, did not receive full protocol therapy (2 patients received only 4 courses because of toxicity; 1 patients received 5 courses but at reduced dosage.) The study group has had a median follow-up of 37 months (range = 2–63 mo.) and median survival is 48 months. There have been 6 disease related deaths and 1 unreleated death in the radiotherapy group; progressive disease was noted at the end of treatment in 3/6 of the disease related deaths. There have been 3 deaths in the chemotherapy group. There is no significant difference in survival between the two groups (log rank chi square (1 df) = 2.27; $P=0.132$). One patient in the chemotherapy group has evidence of recurrent disease. Four patients in the radiotherapy arm have experienced radiation induced complications requiring surgical management. These preliminary results attest to the favourable prognosis of patients with no macroscopic disease following primary surgery. At this stage, there is no significant survival difference between the two groups, although there is long term morbidity from significant radiation induced enteritis. Further follow up is required to determine whether *cis*-platinum will continue to give results comparable with those achieved by radiotherapy in the study of Dembo *et al.*

**Surgery for malignant extradural tumours of the spine**

P.L. Turner, H.G. Prince, J.K. Webb & M.P.J.W. Sokal

*University Hospital, Nottingham, UK.*

Sixty-three patients with malignant extradural tumours of the spine have been treated surgically for spinal cord compression or uncontrolled back pain. Anterior surgery was used in 37 cases, posterior surgery in 22 cases, and in 4 cases combined or staged anterior and posterior decompression.

The medical oncologists were involved in the assessment of the patients on presentation and adjuvant therapy was used post-operatively where this was considered appropriate.

Anterior surgery showed major neurological recovery in 54.5% of cases; only 12.1% of patients remained unchanged; the remaining patients improved neurologically but not sufficient to allow them to walk or to regain bladder function. posterior surgery achieved major neurological recovery in 33.3% of cases; over 44% were unchanged.

Back pain was improved in 73% of those undergoing anterior surgery, and 54% after posterior surgery. The complication rate was similar for the two groups. Five patients died from surgical complications; 36 had died with disseminated carcinoma at the time of review; mean survival in this latter group being 4.1 months. Twenty-two patients are still alive with 17 satisfactory with a mean survival of 14.2 months. Surgery did not give major improvement in the patients presenting with complete paraplegia.

### Endocrine disorders following treatment of brain tumours in childhood

E.A. Livesey, C.G.D. Brook, A.C. Whitton, J.S. Tobias & H.J.G. Bloom

*Middlesex, University College and Royal Marsden Hospitals, UK.*

One-hundred and twelve children (47 girls and 65 boys) treated for brain tumours were studied to assess the prevalence of endocrine disorders other than growth hormone deficiency. All had tumours remote from the hypothalamus or pituitary and were clinically disease free at the time of the study. All had received cranial irradiation, 73 spinal irradiation and 34 adjuvant chemotherapy. Cytotoxic agents were lomustine, vincristine and methotrexate. Mean age at treatment was 6.7 years (1–15). Mean follow-up postradiotherapy was 8.5 years.

Thyroid dysfunction was identified in 29%. Twenty-nine had elevated basal serum TSH concentrations with mean total serum thyroxine of 76.3 $nmol\,l^{-1}$(10–118). Four had secondary hypothyroidism. There was a highly significant association between exposure to chemotherapy and elevated basal serum TSH concentrations, $P<0.001$, and a less significant association with spinal irradiation alone, $P<0.05$.

Primary gonadal dysfunction was found in 17%, 16 girls and 4 boys. Ovarian damage was associated with spinal irradiation and with a younger age at treatment. Primary testicular damage was only associated with chemotherapy particularly lomustine. Hypogonadotrophic hypogonadism was rare (6%).

ACTH deficiency was found in 4 out of 85 children assessed.

### Chemotherapy for thyroid cancer

P.J. Hoskin & C.L. Harmer

*Royal Marsden Hospital, Sutton, Surrey, UK.*

The place of chemotherapy in advanced progressive thyroid cancer is controversial and there are few reported series of its efficacy in this setting.

Twenty-nine patients with primary carcinoma of the thyroid have been treated with sequential chemotherapy regimes using the single agents etoposide, carboplatin, *cis*-platinum or methotrexate and the combination of adriamycin, bleomycin and vincristine (ABC). Indications for chemotherapy were symptomatic recurrent or metastatic disease from follicular, papillary or medullary carcinomas unresponsive to conventional treatment, or advanced anaplastic carcinomas. A total of 60 individual drug exposures have been evaluated in these 29 patients.

Fourteen of 29 patients (48%) responded to one or more agents: 4/22 responded to etoposide, 2/9 to carboplatin, 5/13 to *cis*-platinum, 1/3 to methotrexate and 5/13 to ABC. One complete response was seen following etoposide. Significant drug toxicity occurred in 8 patients. Mean survival in responders was 19 months and in non-responders 5.4 months.

Etoposide, carboplatin, *cis*-platin and ABC appear to be active in advanced thyroid cancer. Useful palliation and improved survival may be achieved in responders.

### The effects of dexamethasone in patients with brain tumours

R.P. Beaney, K.L. Leenders & D.J. Brooks

*Queen Elizabeth Hospital, Birmingham & National Hospital for Neurological Diseases, London, UK.*

Dexamethasone is thought to exert its beneficial effect by reducing perifocal oedema in patients with brain tumours. Clinical improvement, however, commonly occurs before there is any detectable decrease in perifocal oedema. Recent magnetic resonance imaging studies failed to show any decrease in peritumour oedema after the administration of dexamethasone, despite obvious clinical improvement. We set out to see if there was any circulatory cause for this improvement.

Using positron emission tomography we measured regional cerebral blood flow (rCBF) and regional cerebral blood volume (rCBV) in 10 brain tumour patients within 5 days of starting dexamethasone. Regional CBF and rCBV both decreased in a coupled fashion. The mean decrease in rCBF for the whole brain was approximately 15% and the mean decrease in rCBV for the whole brain was 13%.

We think that the initial beneficial effect of dexamethasone is partly mediated through vasoconstriction of cerebral vasculature. Glucocorticoids may cause vasoconstriction by inhibiting the release of prostaglandins, e.g. prostacyclin (a potent vasodilator) from vascular endothelial cells. The magnitude of the clinical response, however, depends very much on the brain tissue compliance of individual patients.

### Thyroglobulin in the follow up of differentiated thyroid cancer

A.M. Cassoni & C.L. Harmer

*The Royal Marsden Hospital, Sutton, Surrey, UK.*

The results of a prospective study on the use of thyroglobulin (Tg) in the follow up of patients with differentiated thyroid cancer are presented. Those studied are a cohort of patients seen for the first time or at follow up after effective thyroid ablation, between June 1978 and December 1984. Of 178 patients, 170 had undergone total or subtotal thyroidectomy and all had received ablative iodine. All were examined regularly, had diagnostic $^{131}I$ scans 6–12 monthly for at least 2 years and had Tg levels measured while taking sufficient thyroid hormone to suppress TSH. Tg was assayed by double radioimmunoassay. At initial assay 31 patients had detectable Tg in the presence of proven disease. A further 5 with detectable Tg subsequently developed disease 4 to 20 (median 12) months later. Two patients, while initially disease free with undetectable Tg subsequently developed elevated Tg 24, and 36 months before disease became apparent. No patient has had disease detected by routine scanning in the absence of an elevated Tg, but in 3 early patients with at least one undetectable Tg there was evidence of recurrent or persistent disease. Of 136 patients without disease at any stage, 29 have had detectable Tg on at least one occasion. However, in all but 4 Tg has now been undetectable for at least 2 years. Tg is an effective means of detecting recurrent or persistent differentiated thyroid carcinoma, without the need for stopping suppressive thyroid hormone. In our view $^{131}I$ scanning should generally be reserved for Tg positive patients.

**Radiotherapy dose perturbations caused by amalgam dental fillings**

M.G. Samarasekara & F.R. Hudson

*Physics Department, Mount Vernon Hospital, Northwood, Middlesex, UK.*

When patients receiving radiotherapy treatment to the head and neck region have exposed amalgam dental fillings significant dose perturbations may be caused. These will result in small zones of high and low dosage and can result in significant mucosal reactions.

Dose distributions have been measured for 5 MV and for Co-60 beams using film and ionisation chamber techniques, to identify the magnitude and the range of the effects. The results show the range to be of up to 3 mm and the magnitude of the perturbed dose to lie between 0.8 and 1.8 times the unperturbed dose.

**The use of electrons in the treatment of intraoral cancer at Mount Vernon Hospital**

E.J. Maher, F.R. Hudson & M.G. Samerasekara

*Department of Radiotherapy, Mount Vernon Hospital, Northwood, Middlesex HA6 2RN, UK.*

Recent publications have suggested a role for electrons in the treatment of intraoral malignancies. Standard electron applicators can be relatively simply modified with an extension adaptor with a slanted tip to allow apposition to selected intraoral sites. The technique has been available at Mount Vernon Hospital for the last 3 years.

Initially location devices were prepared by the dental department, using acrylic material, but the effort involved, bulk and lack of final flexibility were limiting factors. Latterly we have used a simpler technique. A thermoplastic mould is prepared with the patient holding a wide open mouth position. A fixing tray is attached to this shell and the intraoral applicator mounted, in its own separate support, on this tray. Warm thermoplastic is used so the clinician can angle the adaptor until satisfied with tumour cover. The adaptor sets in place with no extra intraoral bulk restricting access and a shell set removes the need for extreme delicacy in positioning each day.

The value of the technique depends on careful patient selection. Even with a large case load of intraoral malignancy, only a few patients will be suitable. Over a 3 year period, 20 were initially assessed as suitable and 12 treated, both alone and in combination with external beam radiation. Clinical uses and results will be compared with our experience using intraoral moulds over the last 10 years.

**Radiation therapy in the management of thymomas**

A.W. Fyles & W.J. Simpson

*The Princess Margaret Hospital, Toronto, Canada*

The optimal management of patients suffering from thymoma is controversial. A review of 69 patients seen at The Princess Margaret Hospital between 1958 and 1980 was undertaken to better define the role of radiation and chemotherapy.

Twenty-one patients had non-invasive tumours, 46 had invasive tumours and in two the degree of invasion could not be determined. Myasthenia gravis was present in 19 patients.

Overall survival was 63% at 5 years; relapse-free survival was 49% at 5 years. For patients with non-invasive tumours the 5-year survival was 80% and for invasive tumours 54%.

Significant adverse prognostic factors included the presence of invasion, unresectable tumour and nodal metastases at presentation. The presence of myasthenia gravis did not influence survival.

Tumour recurrence in the mediastinum occurred in 20% of patients with radiation including 50% of those with unresectable tumour. Complete surgical resection, including resection of pleural metastases if present, is the appropriate initial management for patients with thymoma. Post-operative radiation therapy is indicated for invasive tumours and, combined with chemotherapy, is the primary form of management for those that are unresectable.

**A prospective randomised multicentre trial of adjuvant methotrexate in T3 carcinoma of the bladder**

A. Horwich

*Co-operative Urological Cancer Group, Institute of Cancer Research, Sutton, UK.*

Four hundred patients were randomised to receive local treatment (LT) alone, or LT plus methotrexate (LT+MTX) for T3 bladder carcinoma. LT was irradiation (RT) in two phases to 64 Gy in 32 fractions in $6\frac{1}{2}$ weeks, except in some centres where patients ~65 years were treated with pelvic RT to 44 Gy followed by cystectomy. MTX (100 mg m$^{-2}$ i.v. push with folimic acid rescue) was administered weekly for 3 weeks prior to RT, and following LT MTX dose 100 mg (total) q 2 weeks for 3 months then q 4 weeks for 9 months.

Of 360 evaluable patients with F/U more than 6 months there was no significant difference in median survival between 178 patients randomised to LT+MTX and 182 to LT alone (23 *vs.* 17 months, $P<0.1$). The local recurrence rate was 30% for 146 LT+MTX patients and 28% for 151 LT patients. Eighty-five (48%) of 178 LT+MTX patients developed distant metastases compared to 86 (47%) of 182 Lt patients. There was no difference in the incidence of early side effects of treatment. It was concluded that adjuvant methotrexate was not effective in the context of this study.

**The use of serum prostate specific antigen (PSA) estimation in monitoring hormonal therapy for prostatic carcinoma**

D.A. Gillatt, M. Ferro, I. Barnes & P.J.B. Smith

*Bristol Royal Infirmary, Bristol, UK.*

It is widely accepted that advanced prostatic carcinoma will respond to a reduction of circulating testosterone levels in more than 80% of cases. Various methods including bilateral orchidectomy, gonadotrophin analogues and antiandrogens are in use in our unit. Traditionally treatment response is monitored clinically and by measurement of prostatic acid phosphatase (PAP). Acid phosphatase is, however, raised prior to treatment in only 60% of cases and will be of little use on many occasions.

Prostate specific antigen is produced in the cytoplasm of prostatic cells. It can be measured in serum by an immunoradiometric assay. It is raised above the usually accepted upper limit of normal of 10 ng ml$^{-1}$ in 95% of men with metastatic prostatic cancer. Forty-two patients are being followed up with regular PSA measurement following hormonal manipulation for advanced prostatic cancer. Four patients showed no clinical response, in each case PSA continued to rise. The remaining 38 patients showed good clinical response with a fall in serum PSA levels. It is of interest that the rate of fall varied from rapidly to normal within 1 to 3 months to a more gradual fall over 6 months or more.

Serum prostatic specific antigen measurement provides a more sensitive monitor of prostatic carcinoma than either acid or alkaline phosphatase. PSA should be the main tumour marker for prostatic cancer.

### Platinum induced renal damage

C.R. Hamilton & A. Horwich

*Royal Marsden Hospital, Sutton, Surrey, UK.*

Seventeen patients presenting to the Testicular Tumour Unit of the RMH with malignant teratoma were studied to assess the late renal toxicity of 'BEP' chemotherapy (4–6 courses).

All had normal GFR's as assessed by EDTA renal clearance, and no evidence of obstructive uropathy or past medical history of renal disease. GFR was measured before any chemotherapy and before subsequent courses. It was also assessed in these patients who were rendered disease free 8–57 months (median 31 months) after cessation of all chemotherapy (post-treatment GFR). The mean GFR fell from 132 ml min$^{-1}$ (95% confidence limit 123–141) to 107 ml min$^{-1}$ (95% confidence limit 100–114) whilst on treatment. The post-treatment GFR mean was 103 ml min$^{-1}$.

Of the 17 patients, the post-treatment GFR improved by over 10% in 5 patients and continued to fall by more than 10% in 5 patients, the most notable being from a pre-treatment GFR of 157 ml min$^{-1}$ to 116 ml min$^{-1}$ on treatment, to a post-treatment value of 75 ml min$^{-1}$ two years after cessation of chemotherapy.

### Comparative sensitivities of human bladder and testicular germ cell tumours to chemotherapeutic drugs and gamma-radiation *in vitro*

J.R.W. Masters, M.C. Walker, C.N. Parris, A.R. Lehmann, M.L.H. Greene & C.F. Arlett

*Institute of Urology, London and MRC Cell Mutation Unit, University of Sussex, Brighton, UK.*

Adriamycin, *cis*-platin and gamma-radiation sensitivities *in vitro* of continuous cell lines derived from 5 human non-seminomatous testicular germ cell tumours and 5 human transitional cell carcinomas of the bladder were compared. The range of adriamycin concentrations reducing clonogenic cell survival by 70% following continuous exposure for the testicular cell lines was 0.8–7.2 ng ml$^{-1}$, compared with 3.6–18.6 ng ml$^{-1}$ for the bladder cell lines, and the corresponding figures for *cis*-platin were 19–161 and 112–431 ng ml$^{-1}$. *Cis*-platin binding to DNA following exposure to an equimolar concentration was identical in a bladder and a testicular line with a two-fold difference in sensitivity. There was no correlation between population doubling time and drug sensitivity. Extrapolating from the gamma-radiation survival curves, Do values for the bladder lines ranged from 1.6–2.1 Gy, compared with 1.2–1.5 Gy for the testicular lines. DNA synthesis following gamma-radiation was not inhibited in two testicular cell lines to the same extent as that in two bladder lines.

It is concluded that testicular germ cell tumours retain their characteristic sensitivity to cytotoxic agents *in vitro*. Consequently, drug sensitivity probably is an inherent feature of these tumour cells, and not dependent on humoral factors such as blood supply or immunogenicity. Our preliminary data suggest that testicular tumour cells may have a relatively low capacity to repair DNA following exposure to cytotoxic agents.

### Regression of carcinoma of bladder after radical radiotherapy

E.M. Bessell

*The Hogarth Centre of Radiotherapy and Oncology, Nottingham, UK.*

A study of the regression of carcinoma of the bladder after radical radiotherapy was undertaken to determine whether there was any correlation between the rate of regression and the time to recurrence. This information would be useful in predicting which patients might need salvage cystectomy.

Twenty patients with carcinoma of the bladder, treated with radical radiotherapy have been studied so far. The regression of primary bladder carcinoma was measured during and after radical radiotherapy. In 12 patients the regression was determined by transabdominal ultrasound alone and in 8 patients by ultrasound and by computed tomography.

The regression of transitional-cell carcinoma of the bladder after radiotherapy is much slower than for squamous cell carcinoma of the head and neck or for carcinoma of the breast. The range of volume-halving time was 7–105 days (median 39 days). Considerable calcification occurred during the regression of some of these tumours.

### *Cis*-platinum before radical radiotherapy in transitional cell carcinoma of the bladder: Interim report of the West Midlands CRC trial

I.G. Conn

*Queen Elizabeth Hospital, Birmingham for the West Midlands Urological Research Group, UK.*

A prospective randomised trial is in progress in the West Midlands region to assess the value of three courses of *cis*-platinum (*cis*-diaminodichloroplatinum) 100 mg m$^{-2}$ at 3 weekly intervals before radiotherapy (64 Gy/30 fractions) compared with radical radiotherapy alone in invasive transitional cell carcinoma of the bladder. From July 1984 to January 1987, 108 patients have been entered into the trial from 9 centres in the region. Fifty-seven patients have been randomised to receive chemotherapy of whom 39 have completed three courses at full dosage. Reasons for dose reduction or cessation have been low creatinine clearance in 15 and myelosuppression in 6.

The initial response to chemotherapy has been assessed by CAT scan tumour volume measurement before and after 3 courses of *cis*-platinum. Thirty-nine patients have pre- and post-chemotherapy scans of whom 30 have evaluable disease (T3 or T4). Eighteen (60%) have shown a greater than 50% reduction in tumour volume following chemotherapy.

Of 85 patients evaluable, 75 (88%) have completed a full course of radical radiotherapy. No increased toxocity in the chemotherapy group has been noted.

It is hoped that the initial response to *cis*-platinum will be reflected in improved local control and increased survival.

### Ultrasound guided transperineal $I^{125}$ seed implantation for prostatic carcinoma

J.M. Rodriguez[1], M. Halliwell[1], H. Appleby[1], H. Eckert[1], E.C. Whipp[1], A. Fellows[2], K. Durrant[2], R. Belton[2], C. Keen[3], B. Peeling[3], A. Tyler[3], S. Carter[4], N. O'Donoghue[4] & J. Tobias[4]

[1]*Bristol,* [2]*Oxford,* [3]*Newport and* [4]*London, UK.*

Transrectal ultrasound guided implantation has been developed as an alternative to external beam radiotherapy for early prostatic carcinoma. Initial experience is reported from four centres: Bristol Radiotherapy and Oncology Centre, St Woolos Hospital Newport, St Peter's Hospital in association with University College Hospital London, Churchill Hospital, Oxford.

Thorough preoperative staging is performed and meticulous preliminary planning is required to construct a 3 dimensional model of the prostate and to calculate the number of seeds necessary to deliver a matched peripheral dose of 160 Gy. Implantation is carried out under general anaesthesia using a Bruel and Kjaer 1846 transrectal ultrasound scanner with a special grid attachment which allows precise spatial distribution of seeds within the gland.

Thirty-seven patients with early disease ($T_0, T_1, T_2, N_0, M_0$) have been treated; 35 of these were well differentiated tumours. There has been no operative mortality and very little immediate morbidity and late complications are rare, but include impotence (2), incontinence (1) and perineal pain (1). Despite the high radiation dose to the prostate itself, the rectal dose is minimal in contrast to external beam therapy. An early response is commonly seen as a diminution of prostate volume. Follow up is from 4 months to 2 years. Two patients with poorly differentiated tumours have undergone disease progression.

Time in hospital is 3 days contrasting with 5 to 6 weeks for external beam therapy. Transperineal placement of $I^{125}$ seeds is a simple effective treatment for early prostatic carcinoma.

**Multiple fractions per day – pelvic irradiation**

M. Quigley, M. Brada, J. Bradbeer & A. Horwich

*Royal Marsden Hospital and Institute of Cancer Research, Sutton, Surrey, UK.*

With the intention of defining early tolerance of accelerated fractionation in pelvic irradiation a schedule was developed employing multiple fractions per day and gradually decreasing overall treatment time. We report acute reactions in 34 patients treated for palliation of advanced inoperable pelvic malignancies (24 colorectal tumours, 9 bladder/prostatic tumours and one sarcoma). A total dose of 48.6 Gy was given in 3 blocks consisting of 1.8 Gy fractions given 3 times per day for 3 days with at least $3\frac{1}{2}$ hours between fractions. The overall treatment time (OTT) was varied by reducing the interval between the 3 day blocks. Twenty-seven patients had schedule A (OTT = 31 days), and 7 patients schedule B (OTT = 24 days). Acute toxicity was scored using WHO grades. The major acute toxicity was gastro-intestinal (GI); 56% of patients in schedule A had GI toxicity (15% Grade 1, 22% Grade 2, 18% Grade 3, and 0% Grade 4), compared to 72% of patients in schedule B (29% Grade 1, 14% Grade 2, 29% Grade 3 and 0% Grade 4). Only 3 patients had significant skin toxicity (WHO Grade 3) and all had received schedule B. Nine patients had genito-urinary toxicity, which was Grade 1 or 2 in all cases. Both schedules were well tolerated. Patients treated with schedule B had more acute toxicity, but this did not cause delay in treatment or significant distress.

**Bilateral testicular tumours**

M. Mason & A. Horwich

*Radiotherapy Unit, The Royal Marsden Hospital, Sutton, Surrey, UK.*

A retrospective analysis was performed of 35 patients with bilateral testicular germ-cell tumours whose first tumour was treated between 1958 and 1984 in order to define the natural history of the second tumour. There was a history of maldescent in 28% of evaluable patients, infertility in 22%, and both maldescent and infertility in 21%. The distribution of histology of the first tumour (seminoma in 17, teratoma in 11, combined tumours 3) was not significantly different from the distribution in the second tumour (seminoma 15, teratoma 8, combined 7). Additionally, there were 4 patients who presented with synchromous tumours and 2 of these had different histologies in the 2 tumours.

Twenty-nine patients had no treatment to the contralateral testis following their first orchidectomy (abdominal node irradiation in 23 patients, surveillance in 5 patients and node dissection in 1 patient). Two patients were treated with chemotherapy for their first testicular tumour. One patient received four courses of bleomycin, etoposide and *cis*-platinum which he completed $4\frac{1}{2}$ years before the presentation of his second tumour. He is disease-free one year after his second orchidectomy. The other received 6 courses of vinblastine and bleomycin which he completed 9 months before the presentation of his second tumour. He died with widely disseminated disease 14 months after his second orchidectomy despite further chemotherapy. Testicular biopsy studies suggest that tumours are unlikely to arise in the absence of carcinoma *in situ* and this study would suggest that chemotherapy does not prevent development of all testicular tumours.

**Modification in cardiac function in rats after single doses of anthracyclines**

T.K. Yeung, R.H. Simmonds & J.W. Hopewell

*Research Institute, Churchill Hospital, Oxford OX3 7LJ, UK.*

Modification to the cardiac output in Sprague Dawley rats was assessed following the intravenous administration of single doses of epirubicin (2–10 mg kg$^{-1}$). Cardiac function was measured using an isotope dilution technique at 4-weekly intervals for up to 20 weeks. After a sharp initial decline in cardiac function in drug treated animals (phase I), values remained persistently depressed (phase II) indicating the irreversible nature of anthracycline-associated cardiac lesions. The duration of phase I varied from 4 to 12 weeks depending on dose. In phase II cardiac output values were relatively stable for most animals and deterioration in heart function was usually only in those rats which had shown a >40% reduction in cardiac function in phase I. There were no statistically significant differences between the mean heart rate measured in drug treated and age-matched control animals. This suggested that the reduction in cardiac function was due to the loss of contractile elements in the cardiac muscle. The 50% incidence dose ($ED_{50}$) for a >30% or a >50% reduction of cardiac output, 12 weeks after drug treatment, was $3.34 \pm 0.4$ mg kg$^{-1}$ and $4.93 \pm 0.43$ mg kg$^{-1}$ respectively.

After the administration of single doses of adriamycin, the modification in cardiac function followed the same time course to that after epirubicin. However, adriamycin showed a greater acute effect and cardiotoxicity. Adriamycin was found to be twice as cardiotoxic as epirubicin and this was independent of the drug dose and hence the level of damage in the heart.

**Management of radiation enteritis – An algorithmic guide**

P.L. Zentler-Munro & E.M. Bessell

*St. George's Hospital Medical School, London; and Hogarth Centre, Nottingham, UK.*

The management of diarrhoea and steatorrhoea due to radiation enteritis can prove troublesome. Several pathophysiological abnormalities can be involved, each requiring specific therapy. Choosing the treatment most likely to succeed in each case therefore depends on identifying which mechanism is involved. Gastroenterologists have recently developed several non-invasive diagnostic techniques which can be used to pinpoint the precise cause in each case simply and rapidly. Some of these tests – to identify di- and monosaccharide malabsorption, bile acid deconjugation, bile acid malabsorption, gut hurry and mucosal inflammation – may not be familiar to radiotherapists.

An algorithm has therefore been devised in which each of the tests is arranged so as to arrive at a diagnosis with as few procedures as possible, but it has yet to be formally tested.

**The effect of WR-2721 and antacid therapy on gastric distension caused by a platinum (i.v.) cytotoxic drug**

B. Jones[1] & M.G. Stone[2]

[1]*Radiotherapy Department, London Hospital; and* [2]*Richard Dimbleby Department, St. Thomas Hospital, UK.*

Many clinical studies have shown that the thiophosphate radioprotective compound WR-2721 has emetic side effects and does not protect against the emetic effects of cytotoxic drugs and radiation. This may be due to the known degradation of WR-2721, forming toxic metabolites, at low pH values as found in gastric juice. The effect of neutralisation of stomach pH was tested in an animal model.

$C_3H$ mice were given WR-2721 (400 $kg^{-1}$ i.p.) both with and without antacid treatment (oral sodium bicarbonate and cimetidine) 1 h before and 24 h following the i.p. administration of the platinum (i.v.) compound CHIP which causes marked gastric (volume) distension at doses between 5 $mg\,kg^{-1}$ and 90 $mg\,kg^{-1}$ (LD 50/30 = 65 $mg\,kg^{-1}$). WR-2721 caused increased distension at lower CHIP doses (5–20 $mg\,kg^{-1}$). At higher CHIP doses (30–90 $mg\,kg^{-1}$) the following average reductions in gastric distension were seen: WR-2721 alone −27%; WR-2721 + antacids −35%; antacids alone −33%.

Antacid treatment alone was as effective as WR-2721 at high CHIP doses but did not cause enhanced distension at lower doses. A reduced food and water intake seen with WR-2721 and antacids did not account for the low dose results but this mechanism may be operative at higher doses although a direct effect on stomach pH *in vivo* has not been excluded. A clinical trial utilising antacid drugs in situations involving a high risk of emesis is suggested.

**Late normal tissue damage following intra-arterial adriamycin plus radiotherapy and conservation surgery for soft tissue sarcomas**

M. Mason, C. Harmer & G. Westbury

*Sarcoma Unit, Royal Marsden Hospital, London, UK.*

The use of intra-arterial adriamycin as an adjunct to radiotherapy and limb conserving surgery has previously been described in an attempt to improve local control rates without amputation in the primary treatment of soft tissue sarcoma (STS). The late normal tissue damage that was described following such an approach raised concern about its toxicity, and prompted a pilot study, which is presented here, in which late normal tissue damage was specifically assessed. Ten patients with STS were treated by this trimodality approach using conventionally fractionated radiotherapy; their late normal tissue damage was compared with that in 10 patients recently treated by radiotherapy and conservation surgery without adriamycin. No significant difference in late morbidity was observed between the two groups. This approach appears safe provided conventional fractionation is employed. The late normal tissue damage seen in previous series might be accounted for by the unconventional radiotherapy fractionation employed. It remains to be proven that the addition of intra-arterial adriamycin to surgery and radiotherapy confers any benefit in terms of local control or survival.

**A randomized prospective observer blind trial of E45 cream in the early skin reaction following post-mastectomy chest wall radiotherapy**

M.H. Robinson

*Royal Marsden Hospital, London, UK.*

Management of the early skin reaction to orthovoltage radiotherapy is controversial. There are proponents and opponents of treatment with medicated and non-medicated skin creams. Use of a new formulation of E45 cream was therefore studied in patients who received post-mastectomy radiotherapy to the chest wall and/or the axilla and supraclavicular region (a mid-plane dose of 38 Gy in 10 fractions over 28 days given 3 times a week).

Patients were randomised to apply E45 cream twice daily for 28 days or not at all after radiotherapy (day 0). The doctor assessed the skin reaction with 5 point scales for severity of reaction, redness, dryness, itchiness and soreness on days 0, 14 and 28. Patients kept daily records. At day 14, the physician determined whether patients needed treatment for the skin reaction. If so, this was dispensed. The group using the cream previously continued to do so.

Forty-two patients entered. Groups were comparable in the development of the skin reaction. Significant differences ($P < 0.01$) were found in favour or E45 cream for skin dryness in patients with constant and changed therapy. Skin soreness at day 28 was less ($P < 0.02$) in untreated patients assigned to use E45 cream from day 14. Patients therefore benefit from E45 cream application as therapy for the acute skin reaction to radiotherapy.

**Endoscopic laser treatment for tracheobronchial tumours**

P.J.M. George[1], C.P.O. Garrett[2] and M.R. Hetzel

*Departments of* [1]*Thoracic Medicine and* [2]*Anaesthetics, University College Hospital, London WC1, UK.*

Although endoscopic laser treatment is an established palliative treatment in patients with advanced lung cancer, little is known of its effects on lung function and quality of life. We have treated 105 patients with a neodymium YAG laser under general anaesthesia. The indications for treatment were either breathlessness, due to partial or complete airway obstruction by intraluminal tumour (95 patients), or haemoptysis (10 patients). Assessments of breathlessness, wellbeing, performance status and lung function were made before and after treatment.

In patients with partially obstructed airways ($n = 79$), an overall symptomatic improvement was noted in 59 (75%), while peak flow and/or spirometry rose by at least 20% in

51 (65%). Patients with tracheal and main carinal tumours ($n=49$) reported the most impressive symptomatic gains and exhibited the most marked increases in peak flow (mean rise of 57%; $P<0.001$) and $FEV_1$. In patients with partial obstruction of more peripheral airways (mainstem and lobar-bronchi; $n=30$), symptom scores and lung function also improved significantly ($P<0.01$). In patients with complete endobronchial obstruction ($n=16$), treatment was successful in 9 (56%); re-expansion was associated with improved symptom scores and increases in FVC, the best results being seen in patients with main bronchial obstruction ($P<0.05$). Haemoptysis was completely abolished in 8 patients (80%); the period of relief ranged from 3 weeks to 4 months.

We conclude that endoscopic laser treatment provides significant relief from haemoptysis and breathlessness in selected patients, and that the response in patients with breathlessness varies according to the level of obstruction.

### Management of severe malignant pelvic pain by intrathecal opiates, administered by an implanted intrathecal catheter and subcutaneous reservoir

M. Powell

*Department of Neurosurgery, The Middlesex Hospital, London W1, UK.*

Intrathecal opiates have been administered to 6 patients with severe pain from disseminated malignant disease resistant to oral and intramuscular analgesia. The pain was mainly pelvic in origin.

The system consists of standard components of a lumbo-peritoneal shunt (approximately £280). Implanted under general anaesthetic a 4FG catheter is introduced by a Tuohy needle into the lumbar sac and connected to a 3.5 ml subcutaneous reservoir over the ribs, allowing percutaneous intrathecal injections. In 5 cases the patient could stop all alternative means of opiate administration, to be replaced by either OM or bd diamorphine 5–15 mg in various dilutions of saline (usually 10 mg in 7 ml). Three patients returned home, diamorphine being given by GP, district nurse and spouse. Two (with colostomies) required the systems removal for skin commensal meningitis at 6 and 8 weeks. The third patient's reservoir was replaced because of multiple puncture leakage at 10 weeks. The remaining patients died relatively pain-free in hospital.

A similar system was placed epidurally following the removal of the first for meningitis. It was not successful. An alternative subcutaneous system (Cordis Secor TM cost £490) is being evaluated at present. It has a measured dose pump, patient administered on demand from a reservoir holding approximately 1 month's supply of morphine.

In conclusion, a simple, cheap and effective system for pain control is presented, best for short-term use and contra-indicated in colostomy patients in community care. A better but more expensive long-term system is under evaluation.

### The role of computed tomography (CT) in selecting patients for hindquarter amputation (HQA)

R.M. Warkins & J.M. Thomas

*Westminster Hospital, London, UK.*

The suitability of patients with sarcomas of the thigh, buttock or pelvis for HQA is usually determined by clinical examination. Our aim was to evaluate the role of CT in assessing their operability.

Ten patients were referred for HQA. Patients were considered unsuitable for HQA if on surface examination malignant disease extended into the perineum, across the sacro-iliac joint or above the inguinal ligament. Buttock tumours palpable on pelvic examination were also considered inoperable. Tumours were considered inoperable if CT showed buttock disease extending through the greater sciatic notch, the psoas muscle was involved above the inguinal ligament, the disease crossed the sacro-iliac joint or the perineal structures were involved.

Five patients thought suitable for HQA on clinical assessment had no excluding features on CT. One refused surgery; 4 undergoing HQA had no malignant disease at the resection margins. In a further patient, considered suitable for amputation, CT suggested that wide excision of the tumour with limb-preservation was feasible. After clinical examination 4 patients were considered unsuitable for HQA; CT confirmed inoperability in each case.

The results of clinical assessment are usually confirmed by CT which provides an objective means of selecting patients for HQA. CT may also identify those patients suitable for limb-preservation.

### Lymphokines in malignant melanoma

D.C. Dumonde[1], M.S. Pulley[1], J.M. Edwards[2] and A.R. Timothy[3]

*Departments of [1]Immunology and [3]Radiotherapy and [2]Surgical Unit, St. Thomas' Hospital, London, UK.*

Lymphokines are non-antibody proteins generated by lymphocyte activation; current evidence implicates lymphokines in effecting both specific and non-specific immunity. The rationale for administering lymphokines in malignant melanoma is to assist host defence mechanisms against tumour spread and to help maintain the integrity of the immune system. In this paper we review current approaches to the therapeutic investigation of lymphokines in malignant melanoma.

Rosenberg and colleagues have reported that i.v. administration of high dose interleukin-2 (IL-2) both alone and in combination with autologous lymphocytes activated *ex vivo* with IL-2 (LAK cells) has resulted in regression of melanoma. In our preliminary work we have used buffy-coat interleukin (BC-IL), containing several lymphokine activities including IL-2, which has so far been well tolerated. Endolymphatic administration of lymphokines and LAK cells may be particularly appropriate in melanoma which spreads primarily by lymphatic pathways. We have demonstrated clinical, radiological and histological evidence of lymph node activation following administration of BC-IL by this route.

Our experience and that of other investigators suggests that the administration of lymphokines may be of value in malignant melanoma. Protocols need to be designed to explore methods of administration in patients with disease of different stages and the administration of lymphokines as an adjunct to other forms of treatment. The monitoring of white-cell function in these patients may yield important information in the design of these protocols.

### Hyperthermia sensitivity of human melanoma and neuroblastoma cells grown as multicellular spheroids

R.D. Jones, I. Berry, R. Hamlet & T.E. Wheldon

*Glasgow Institute of Radiotherapeutics and Oncology, UK.*

Malignant melanoma and neuroblastoma are cancer types which differ markedly in their clinical radiosensitivity. Interest exists in possible treatment of radioresistant tumours by other modalities such as hyperthermia. For melanoma, one therapeutic procedure involves combined use of hyperthermia with melphalan via the technique of isolated limb perfusion. Experimental studies of heat sensitivity of melanoma and other tumour types may be of clinical relevance. Multicellular spheroids derived from melanoma and from neuroblastoma were subjected to various regimes of heat treatment in the temperature range 39–45°C. Spheroid responses were quantified by analysis of regrowth curves to yield estimated cell survival data. Arrhenius plots were constructed and activation energies calculated. These studies may be useful in relating radiosensitivity to heat sensitivity and in the design of clinical studies in which hyperthermia is a therapeutic component.

### The prospects for radioimmunotherapy of occult metastases using antibody-targeted $^{131}I$

T.E. Wheldon, J.A. O'Donoghue, T.E. Hilditch & A. Barrett

*Glasgow Institute of Radiotherapeutics and Oncology, UK.*

Radioimmunotherapy entails selective irradiation of tumour cells by delivery of radionuclides conjugated to antibodies. Mathematical models studies show that radioimmunotherapy using $^{131}I$ is not yet capable of delivering radical doses to occult metastases. This is because the limited specificity of available antibodies results in high doses to normal tissues, especially bone marrow. The analysis suggests, however, that radioimmunotherapy followed by bone marrow rescue should be an effective treatment. Logistic considerations imply that the optimal strategy might be a combination of radioimmunotherapy, external-beam TBI and marrow rescue. This combination strategy presently seems the most promising appraoch to use of radioimmunotherapy as treatment for occult metastases.

### Manipulation of tumour oxygenation to increase the potency of bioreductive radiosensitizers

G.E. Adams & I.J. Stratford

*MRC Radiobiology Unit, Chilton, Didcot, UK.*

The vasocactive drug hydralazine can cause substantial changes in blood flow to murine tumours. One result of these changes in the induction of close to 100% hypoxia in subcutaneous tumours, which lasts for nearly 2 h following an i.v. injection of $5\,mg\,kg^{-1}$. Induction of tumour hypoxia could be therapeutically beneficial, particularly when used with radiation and the nitroimidazole radiosensitizers. This is demonstrated with results obtained using the KHT tumour *in vivo* when the following treatment schedule is employed:

$$\text{Sensitizer} \rightarrow 60\,\text{min} \rightarrow \text{X-rays} \rightarrow 1\,\text{min} \rightarrow \text{hydralazine}.$$

Such a strategy will first exploit the radiosensitizing properties of the nitroimidazole, then after irradiation administration of hydralazine will induce tumour hypoxia and allow expression of the differential toxicity towards hypoxic cells known to occur with agents such as RSU 1069 and misonidazole.

This approach results in a substantial increase in the apparent efficiency of misonidazole and RSU 1069 as radiosensitizers. For example, the enhancement of tumour cell killing achieved by $100\,mg\,kg^{-1}$ misonidazole when used in combination with hydralazine is equivalent to that obtained by $1000\,mg\,kg^{-1}$ misonidazole alone.

### Trials data collection by portable microcomputer

S.N. Larson

*CRC Clinical Trials Unit, Birmingham, UK.*

Data collection for large trials usually involves three stages: (1) recording data in the clinical notes; (2) filling in study forms; (3) transferring data to the unit's computer system for analysis.

We are streamlining this process using an easily portable microcomputer and software developed in-house, allowing direct data collection in the clinic and transfer to our main unit computer system for detailed analysis. We use an IBM-compatible Datavue 25 portable microcomputer with 768 kilobytes of random access memory and twin built-in 3.5 inch disc drives, each with 720 kilobytes of storage. The software is written in Psion Archive, an advanced programmable database language. it is designed to be menu driven, suitable for users with no computer expertise. Data collected at the clinic are stored as a database file, with one record of 139 fields per patient. String fields are used with each data item for a particular visit concatenated to the existing string for that field. Routines have been developed which allow data to be 'sliced' out of this string appropriately. As the trials in question were under way before the computer was acquired, a facility is provided to collect data retrospectively from clinical notes.

Further software allows data to be presented as a standardised printout of all data for a patient, or data for one visit. This facilitates subsequent entry of data into the unit VAX minicomputer. The software already developed involves ~70 kilobytes of programming. We are now developing protocols for direct file transfer from the Datavue to the VAX via its integral serial interface.